# Vitamins and minerals and their role in cancer: a comprehensive review

**DOI:** 10.3389/fnut.2025.1686777

**Published:** 2026-01-12

**Authors:** Mattia Garutti, Marco de Scordilli, Martina Alberti, Roberta Mazzeo, Anna Michelotti, Sofia Fagioli, Alessandro Del Conte, Amanda Casirati, Riccardo Caccialanza, Enrico Bartolini, Fabio Puglisi

**Affiliations:** 1 Centro di Riferimento Oncologico di Aviano (CRO), IRCCS, Aviano, Italy; 2 Department of Medicine, University of Udine, Udine, Italy; 3 Department of Oncology, University Hospital of Udine, ASUFC, Udine, Italy; 4 Pediatric Endocrinology Unit, Department of Pediatrics, IRCCS Istituto Giannina Gaslini, Genoa, Italy; 5 Department of Neuroscience, Rehabilitation, Ophthalmology, Genetics, Maternal and Child Health, University of Genoa, Genoa, Italy; 6 Clinical Nutrition and Dietetics Unit, Fondazione IRCCS Policlinico San Matteo, Pavia, Italy; 7 Department of Oncology and Hematology-Oncology, University of Milan, Milan, Italy; 8 Enrico Bartolini Restaurant, Milan, Italy

**Keywords:** oncology, nutrition, culinary medicine, vitamins, minerals, micronutrients, cancer prevention, supplements

## Abstract

Cancer remains a leading cause of morbidity and mortality worldwide, influenced by genetic, environmental, and lifestyle factors, including nutrition. This review explores the role of vitamins and minerals in cancer prevention and management, highlighting their critical functions in immune response, DNA synthesis, cellular repair, and antioxidant defense. Vitamins such as A, C, D, E, and the B-complex group, along with minerals like calcium, magnesium, and zinc, are essential for maintaining health and managing oncologic diseases. Cancer patients are often at risk of micronutrient deficiencies due to disease and treatment-related factors; addressing these deficiencies through dietary interventions or supplementation may enhance immune function, reduce treatment-related side effects, and improve overall quality of life. In this review, we comprehensively discuss the biology and physiology of vitamins and minerals with a specific focus on cancer while providing a practical overview of recognizing and managing deficiencies. Furthermore, aligned with the principles of culinary medicine, we have developed a set of recipes for patients and caregivers to manage hypokalemia and hypocalcemia, which are particularly common in clinical practice, aiming to offer useful tools for clinicians and dietitians.

## 1 Introduction

Cancer remains a leading cause of morbidity and mortality worldwide, affecting millions of individuals each year (1). Estimates from the International Agency for Research on Cancer (IARC) report close to 20 million new cases of cancer in 2022, with an expected number of 35 million new cases by 2050 (2). As a multifactorial disease, its development and progression can be influenced by various genetic, environmental, and lifestyle factors, including diet and nutrition (3).

Vitamins and minerals, essential micronutrients required for numerous physiological functions, play a critical role in maintaining health and preventing diseases (4). Vitamins such as A, C, D, E, and the B-complex group, along with minerals like iron, calcium, magnesium, and zinc, are vital for immune function, DNA synthesis, cellular repair, and antioxidant defense mechanisms (4). In oncology, recognizing and correcting micronutrient deficiencies is paramount. Indeed, cancer patients are often at risk of these deficiencies due to disease- and treatment-related factors such as reduced food intake, malabsorption, and increased metabolic demands (5). Identifying and addressing these deficiencies could significantly impact patient outcomes by improving immune function, reducing treatment-related side effects, and enhancing overall quality of life (5).

Much evidence is available in the literature regarding the potential protective effect of micronutrients in reducing cancer risk. For example, calcium deficiency has been reported as possibly associated with an increased risk of colorectal or breast cancer (6–9). Chromium has been classified as carcinogenic by IARC, and a correlation between serum levels and the risk of lung cancer has been reported (10). An excess of iron is associated with an increased risk of cancer, particularly hepatocellular carcinoma (HCC) (11). The available evidence is less robust and sometimes controversial for other micronutrients, such as vitamin C (12) or the B vitamins (13–16).

The relationship between vitamin and mineral supplementation and cancer is still a highly debated topic, considering the widespread use of various supplements and the often-conflicting results reported in the literature (17). For instance, Lim et al. reported a slightly higher overall risk of prostate cancer, lung cancer, and leukemia in men consuming one or more multivitamins daily compared to non-users, and a higher risk of oropharyngeal cancer in women, while daily multivitamin use was inversely associated with the risk of colon cancer (18).

In this review, we comprehensively discuss the biology and physiology of vitamins and minerals with a specific focus on cancer (Tables 1–4), aiming to highlight their potential role in cancer prevention based on the available evidence, while also emphasizing the importance of addressing deficiencies during oncologic treatment by providing a practical overview to facilitate their recognition and management.

**Table 1:** Overview of vitamins in a clinical setting.

**Element**	**RDA/AI^a^ (USA)**	**Causes of deficit**	**Symptoms**	**Food sources**
Vit. A	M^b^: 900 μg. F^b^: 700 μg (4).	Malabsorption syndromes, oncological or bariatric surgery.	Fatigue, xeropthalmia, night blindness, dry skin and hair, infections, infertility (20).	Dairy products, eggs, fish, meat, yellow/orange/red fruits and vegetables.
Vit. D	M and F (< 70): 600 IU (15 μg). M and F (>70): 800 IU (20 μg) (4).	Malabsorption syndromes, oncological surgery, hepatic/renal insufficiency, aromatase inhibitors.	Fractures, rickets (children) and osteomalacia (adults) (46).	Eggs, fish, mushrooms.
Vit. E	M and F: 15 mg (4).	Malabsorption syndromes, oncological surgery, cholestatic liver disease, genetic disorders.	Infections, retinopathy, peripheral neuropathy, ataxia, hemolysis, brown bowel syndrome (78).	Plant oils, nuts, seeds, fruits and vegetables.
Vit. K	M: 120 μg. F: 90 μg (4).	Malabsorption syndromes, oncological surgery, total parenteral nutrition, antibiotics.	Bleeding (mucosal, melena, hematuria), skin bruises, fractures (92).	Green vegetables, dried plums, kiwis, pistachio nuts.
Vit. C	M: 90 mg. F: 75 mg (4).	Restricted diet, malabsorption syndromes, smoking, alcohol abuse, systemic inflammation.	Fatigue, arthralgias, anemia, scurvy (115).	Citrus fruits, arugula, broccoli, currants, kiwis, papayas, peppers, strawberries.
Vit. B1 (thiamine)	M: 1.2 mg. F: 1.1 mg (4).	Low dietary intake, malabsorption syndromes, refeeding syndrome, diuretics, alcohol abuse, systemic inflammation.	Wernicke-Korsakoff syndrome and Wernicke's encephalopathy, beriberi (128–130).	Cereals, eggs, legumes, meat, oat meal, whole rice, yeast.
Vit. B2 (riboflavin)	M: 1.1–1.3 mg. F: 0.9–1.1 mg (4).	Oncological surgery, CT and anticancer treatments causing diarrhea and vomiting, cachexia.	Fatigue, swollen throat, ocular manifestations, mucocutaneous lesions, depression (147–150).	Dairy products, eggs, green vegetables, meat, yeast.
Vit. B3 (niacin)	M: 1.6 mg NE/MJ. F: 1.3 mg NE/MJ (4)	Restrictive diet, malabsorption syndromes, chronic alcoholism, carcinoid syndrome, isoniazid, Hartnup disease.	Pellagra (175).	Fish, legumes, meat, mushrooms, peanuts, potatoes, wheat germ, whole grain.
Vit. B5 (pantothenic acid)	M and F: 5 mg (4).	Severe malnutrition, malabsorption syndromes.	Fatigue, malaise, weakness, numbness, concentration difficulties, paresthesia, muscle cramps, nausea, vomiting, abdominal cramps (198–200).	Dried mushrooms, egg yolk, legumes, offal, yeast.
Vit. B6 (pyridoxine)	M and F (14–50 y^c^): 1.3 mg. M (>50 y): 1.7 mg. F (>50 y): 1.5 mg (4).	Malnutrition, malabsorption syndromes, parenteral nutrition, alcoholism, pyridoxine-interfering drugs, hemodialysis.	Seborrheic dermatitis, glossitis, angular cheilitis, confusion, neuropathy, convulsions, depression, anemia, neutropenia, lymphopenia (198–200).	Avocados, bananas, chickpeas, meat, lentils, nuts, potatoes, wheat germ, spinach, yeast.
Vit. B7 (biotin)	M and F (14–18 y): 25 μg. M and F (>18 y): 30 μg (4).	Severe malnutrition, malabsorption syndromes, GI tract bacterial imbalances (antibiotics, IBD^d^), parenteral nutrition, concomitant medications, alcoholism.	Alopecia, perioral dermatitis, red rash around orifices, hypotonia, hyposthenia, numbness, poor concentration, nausea, vomiting, weight loss, depression (206, 207).	Bananas, egg yolk, liver, nuts, oranges, whole grain, yeast.
Vit. B9 (folate)	M and F: 400 μg (4, 173).	Malnutrition, malabsorption syndromes, alcoholism, MTHFR polymorphism, drugs interfering with biosynthesis of THF.	Fatigue, muscle weakness, infertility, increased risk of CV^e^ disease, cognitive impairment, dementia, depression, mouth sores or ulcers, macrocytic anemia (214, 215).	Dairy products, green vegetables, kiwis, legumes, lemons, nuts, offal, oranges, strawberries, whole grain, yeast.
Vit. B12 (cyanocobalamin)	US: M and F: 2.4 μg. EU: M and F: 4 μg (4, 173, 226, 227).	Pernicious anemia and atrophic gastritis, vegan diet, malabsorption syndromes, alcoholism, GI surgery, pancreatic insufficiency, PPI^f^, metformin.	Fatigue, weakness, dizziness, neurological deterioration, glossitis, weight loss, infertility, megaloblastic anemia, decreased platelet and white cell count, mania, psychosis (228).	Dairy products, eggs, fish, meat.
Choline	M: 550 mg. F: 425 mg (4)	Malabsorption syndromes, total parenteral nutrition.	Accelerated atherosclerosis, CV diseases, muscle damage, MASLD^g^, neurological disorders (253, 254).	Eggs, fish, legumes, meat, nuts, spinach, wheat germ.

Please see the text for accurate references for each finding.

^a^RDA/AI, Recommended Daily Allowance/Adequate Intake.

^b^M, male; F, female.

^c^y, years.

^d^IBD, inflammatory bowel disease.

^e^CV, cardiovascular.

^f^ PPI, proton pump inhibitors.

^g^ MASLD, metabolic dysfunction-associated steatotic liver disease.

**Table 2:** Overview of minerals in a clinical setting.

**Element**	**RDA/AI^a^ (USA)**	**Causes of deficit**	**Symptoms**	**Food sources**
Calcium	M^b^ (19–70 y^c^): 1,000 mg. M (>71 y): 1,200 mg. F^d^ (19–50 y): 1,000 mg. F (>51 y): 1,200 mg (4, 265)	Malabsorption syndromes, intestinal resection, neck surgery or radiation, vit. D deficiency, renal impairment, hypomagnesemia, low albumin levels.	Increased neuromuscular excitability (Chvostek and Trousseau signs), tetany, seizures, arrhythmias (266).	Dairy products, dark green vegetables, egg yolk, fish, legumes, nuts.
Phosphorus	M and F: 700 mg (4).	Malnutrition, CT- or tumor-induced anorexia, chronic diarrhea, aluminum or magnesium antiacids, refeeding syndrome, antineoplastic drugs (ifosfamide, cisplatin, TKI^e^, ALKi, mTORi, FGFRi).	Muscle weakness, rhabdomyolysis, fatal arrhythmias, confusion, anorexia, rickets (children) and osteomalacia (adults), ataxia, paresthesia, hemolysis, anemia (4, 406).	Almonds, beans, cashews, cocoa powder, dairy products, liver, pistachio nuts, quinoa, soy, spelt.
Magnesium	M (19–51 y): 400-420 mg. F (19–51 y): 310–320 mg (4, 290).	Malabsorption syndromes, GI^f^ resection, vomiting, diarrhea, diabetes mellitus, loop diuretics, PPI^g^, antiacids, antibiotics (aminoglycosides), antineoplastic drugs (cisplatin, cetuximab, panitumumab).	Fatigue, weakness, tetany, nystagmus, tonic-clonic seizures, arrhythmias, delirium (291).	Bananas, dark chocolate, figs, leafy vegetables, legumes, millet, peaches, pears, nuts, prawns.
Sodium	M and F: 1,500 mg (1,300 mg after 50 y) (4, 318).	GI losses, diuretics, hemorrhages, adrenal insufficiency, ACTH deficit, severe hypothyroidism, paraneoplastic SIADH^h^.	Nausea, vomiting, headache, confusion, lethargy, coma (319).	Canned fish, ham, ketchup, loaf bread, margarine, miso, oysters, seasoned cheese, stock cubes.
Potassium	M (14–18 y): 3,000 mg. M (>19 y): 3,400 mg. F (14–18 y): 2,300 mg. F (>19 y): 2,600 mg (4, 332).	Anorexia, malabsorption syndromes, ectopic ACTH, drugs (thiazide diuretics, insulin, granulocyte growth factor, glucocorticoids), antineoplastic drugs (cisplatin, ifosfamide, anti-EGFR agents, mTOR inhibitors, eribulin, abiraterone).	Fatigue, muscle weakness, rhabdomyolysis, constipation, impairment of cardiac contractility (4, 332).	Almonds, fruits, legumes, meat, nuts, vegetables.
Chloride	M and F (14–50 y): 2.3 g. M and F (51–70 y): 2 g. M and F (>71 y): 1.8 g (4, 335).	Diarrhea, vomiting, COPD^i^, chronic respiratory acidosis, diuretics, glucocorticoids, ectopic ACTH.	Dehydration, fatigue, shortness of breath, confusion, nausea, vomiting (4, 335).	Mainly cooking salt; also celery, lettuce, olives, rye, seaweed, tomatoes.
Iron	M: 8 mg. F (18–50 y): 18 mg. F (>50 y): 8 mg (4, 354).	Excessive alkalinization of GI tract (also antiacid drugs, such as H2 receptor blockers, PPI), malabsorption syndromes, rapid transit time, absence of digestive juices.	Anemia, fatigue, pallor, increased infective risk (354).	Heme iron: meat and animal food. Non-heme iron: legumes, vegetables.
Zinc	M: 11 mg. F: 8 mg (4, 354)	Alkaline pH, antiacid drugs (PPI, H2 receptor blockers), food complexes (phytic acid, oxalate, polyphenols, other minerals, such as calcium and iron), alcoholism, sickle-cell anemia, trauma, malabsorption syndromes, diarrhea, total parenteral nutrition, diuretics.	GI symptoms, lethargy, depression, alopecia, skin alterations, hypogeusia, impaired vision, impaired protein synthesis/immune function/wound healing (4, 354).	Dairy products, eggs, fish, fava beans, hemp seeds, meat, oats.
Copper	M and F: 900 μg (4, 354).	Alkaline pH, antiacid drugs (PPI, H2 receptor blockers), food complexes (phytic acid, other minerals, such as zinc, calcium, and iron), nephrosis, malabsorption syndromes, total parenteral nutrition.	Anemia, leukopenia, muscle weakness and fatigue, skin/hair altered pigmentation, altered immune function, bone and blood vessel/connective tissue alterations, cholesterol metabolism alterations, cognitive deficit (354).	Bran, legumes, mollusks, nuts, offal, seeds, whole grain.
Selenium	M and F: 55 μg (4, 354).	Total parenteral nutrition, diarrhea, malabsorption syndromes.	Poor growth, hair loss, skin pigmentation, muscle weakness and pain, cardiomyopathy, whitened nail beds, altered immune system and anti-inflammatory response (4, 354).	Asparagus, cashews, chia seeds, dairy products, dried apricots, fish, red meat, walnuts.
Chromium	M (18–50 y): 35 μg. M (>50): 30 μg. F (18–50): 25 μg. F (>50): 20 μg (4, 354).	Total parenteral nutrition, antiacid drugs (PPI, H2 receptor blockers), malnutrition (increased catabolism and metabolic needs).	Peripheral neuropathy, insulin resistance and high plasma free fatty acid concentrations, weight loss (4, 354).	Apples, bananas, broccoli, meat, oranges, snow peas, whole grain.
Iodine	M and F: 150 μg (4).	Anorexia, malabsorption syndromes, GI resection, geographical area of living, thyroid surgery or radioiodine therapy.	Fatigue, lethargy, sensitivity to cold, constipation, weight gain, dry skin and hair (4, 391).	Cod, dairy products, eggs, iodized salt, seaweed, tuna.
Manganese	M: 2.3 mg. F: 1.8 mg (4).	Highly restricted diet.	Skin and hair alterations, nausea and vomiting, ataxia and loss of equilibrium, abnormal glucose tolerance, skeletal bone changes, impaired reproductive function, low clotting protein levels (4, 354).	Beetroot, coconut, green vegetables, mint, nuts, oats, pineapple, strawberry, tofu, turmeric, whole rice.
Molybdenum	M and F: 45 μg (4).	Total parenteral nutrition, genetic defects.	Seizures, developmental delay, feeding difficulties, increased blood levels of xanthine, methionine, uric acid (4, 397).	Cheese, legumes, offal, nuts.

Please see the text for accurate references for each finding.

^a^RDA/AI, Recommended Daily Allowance/Adequate Intake.

^b^M, male.

^c^y, years.

^d^F, female.

^e^TKI, tyrosine kinase inhibitors.

^f^GI, gastrointestinal.

^g^PPI, proton pump inhibitors.

^h^SIADH, syndrome of inappropriate antidiuretic hormone secretion.

^i^COPD, chronic obstructive pulmonary disease.

**Table 3:** Overview of vitamins in cancer.

**Element**	**Cancer protective effects (with evidence level)**	**Cancer favoring effects (with evidence level)**
Vitamin A	Promotes cell differentiation, modulates gene expression, immune system enhancement (4). Deficit is associated with MASLD^a^ and possibly higher risk of HCC^b^ (III) (22). Depletion associated with cigarette smoke increases the risk of lung cancer (II) (23). Increased intake reduces the risk of gastric cancer (II) (26), cutaneous squamous cell carcinoma (III) (27), cervical cancer (III) (28), and colorectal cancer (III) (29). All-trans retinoic acid (ATRA) regulates several biological processes (cell proliferation, differentiation, apoptosis) and has been used for years as a targeted therapy in acute promyelocytic leukemia (I-II-III) (30, 36–45).	Supplementation of beta-carotene in smokers increases the risk of lung cancer (I) (25). The expression of retinoic acid receptor is often dysregulated in cancer, and ATRA can also act through non-canonical mechanisms with a protumorigenic role (II-III) (31–35).
Vitamin D	Promotes cell differentiation and immune system enhancement (4). Supplementation reduces the risk and the mortality of advanced cancer (I) (67, 68), in particular lower risk of colorectal cancer (CRC^c^) (I) (57), breast cancer (I-II) (58, 59). Deficiency increases the risk of liver cancer (I) (60). Inhibition of thyroid cancer cell proliferation (III) (62).	Higher serum levels may increase the risk of prostate cancer (I-II) (71, 72). Supplementation may have detrimental effects with impaired VDR signaling (III) (73–77).
Vitamin E	Antioxidant effect, immune system enhancement (4). Anti-tumor activity of δ-tocotrienol on pancreatic cancer cells (II-III) (88–91).	Supplementation may increase the risk of prostate cancer (I) (85).
Vitamin K	Epithelial-mesenchymal transition and Wnt signaling pathway suppression in CRC cells (III) (101). Menaquinone induces apoptotic and non-apoptotic death in solid tumors and leukemia cells (III) (102–104, 106). Increased intake reduces the risk of cancer-related mortality in current/former smokers (II) (99), of pancreatic and lung cancer (II) (97, 100). Inhibitory role in HCC development (III) (107, 108).	Supplementation of menaquinone increases the risk of breast cancer (II) (95).
Vitamin C	Antioxidant effect, immune system enhancement, possibly chemosensitivity enhancement, pro-oxidant anti-tumor effect at high doses, hindering tumor cell glucose intake (III) (117, 118). High plasma levels reduce the risk of gastric cancer (II-III) (119, 120) and GI cancer (I) (121). Increased intake reduces the risk of pancreatic cancer (III) (122).	Unknown.
Vitamin B1 (thiamine)	Immune system regulation (4). High doses may shift tumor metabolism, reducing glycolysis (136, 137, 139). Intake inversely correlated with the risk of colorectal cancer (I) (140), bladder cancer (III) (141), and HR+/HER2-^d^ breast cancer (II) (142).	Might remodulate cell metabolism, at low doses, it may enhance tumor growth by thiamine-dependent enzymes (4, 136–138).
Vitamin B2 (riboflavin)	B2 deficiency causes oxidative damage and altered DNA repair (III) (156, 157).	Transporters are overexpressed in tumor cells (III) (158–160)) and may have a prognostic role (III) (164). High serum levels are associated with a higher risk of pancreatic and CRC cancer (II-III) (161, 162).
Vitamin B3 (niacin)	DNA repair, genomic stability, gene expression modulation (III) (180, 181). Supplementation might reduce the risk of HCC (I) (14).	Sustained NAD production supports cell survival and proliferation (III); may cause breath, confusion, nausea, vomiting (4, 335).
Vitamin B5 (pantothenic acid)	*In vitro* administration of CoA or pantethine increases CD8^+^ T cell anti-tumor activity (192, 193). Supplementation may increase anti-cancer immunosurveillance and possibly the response to immunotherapy (195).	High concentrations may increase metabolic activity through the c-MYC pathway (III) (191).
Vitamin B6 (pyridoxine)	1-carbon group transfer for DNA synthesis and methylation. Low levels may determine aberrations in DNA synthesis, repair, and methylation (III) (203). Blood PLP^f^ levels are inversely associated with the risk of colorectal cancer (I) (203). Supplementation is possibly associated with a lower risk of nasopharyngeal carcinoma (I) (14), pancreatic (I) (14, 407), and breast cancer (I) (14, 408).	Unknown.
Vitamin B7 (biotin)	Unknown.	Unknown.
Vitamin B9 (folate)	Genomic stability, DNA repair (221–223).	Aberrant DNA methylation (221–223). Supplementation might increase the risk of malignant degeneration of colorectal adenomas (I) (15, 220).
Vitamin B12 (cyanocobalamin)	Supplementation may reduce the risk of CRC (II) (244) and the carcinogen-induced oxidative stress in CRC cells (III) (243). Serum levels may play a role in epigenetic regulation in CRC patients (III) (245).	Elevated levels or supplementation are possibly associated with a higher risk of cancer (II-III) (16, 239–241, 246–248).
Choline	Low levels are associated with MASLD/MASH^f^, oxidative stress due to mitochondrial dysfunction, and increased sensitivity to chemical insults, leading to a higher risk of HCC (III) (260, 261). Supplementation might reduce the risk of MASLD-related HCC (III) (262).	High serum levels of choline and altered microbiome choline metabolism might increase the risk of colorectal cancer (I, II) (263, 264).

Level of evidence: I -> meta-analysis or randomized controlled trial, II -> prospective cohort, III -> retrospective cohort or preclinical evidence. Please see the text for accurate references for each finding.

^a^MASLD, metabolic dysfunction-associated steatotic liver disease.

^b^HCC, hepatocellular carcinoma.

^c^CRC, colorectal cancer.

^d^HR+/HER2-, hormone receptor positive/human epidermal growth factor receptor negative.

^e^PLP, pyridoxal 5-phosphate.

^f^MASH, metabolic dysfunction-associated steatohepatitis.

**Table 4:** Overview of minerals in cancer.

**Element**	**Cancer protective effects (with evidence level)**	**Cancer favoring effects (with evidence level)**
Calcium	High intake associated with a lower risk of recurrence of colorectal polyps (I) (274, 275) and of developing colorectal cancers (II) (276–279). High intake of calcium and vit. D might reduce the risk of premenopausal breast cancer (II) (9, 284).	Unknown.
Phosphorus	Unknown.	High levels promote cell cycle progression and lung cancer formation in animal models (III) (288).
Magnesium	Intake might reduce the risk of breast, liver (III) (290), and colon (I-II) (304–306) cancer. Adequate intake might increase survival in breast cancer (II) (307) and colorectal cancer (I-II) (305, 308) patients and is associated with reduced all-cause and cancer-related mortality (I) (309).	Favors cell proliferation and metastatic colonization in preclinical models, and several magnesium transporters are overexpressed in several solid tumors (III) (310–316).
Sodium	Unknown.	High intake of salty food might increase the risk of gastric cancer (III) (318). Intracellular sodium is increased in tumor cells, and voltage-gated sodium channels are implicated in several mechanisms of tumorigenesis (III) (323–331).
Potassium	Unknown.	Many potassium channels are involved in cancer proliferation and are expressed in different types of tumors (III) (333).
Chloride	Unknown.	High intake of salty food might increase the risk of gastric cancer (III) (318). Several chloride transporters are overexpressed in solid tumors (III) (345–352)
Iron	Iron excess can promote ROS^a^ formation (lethal for cancer cells) (III) (11). Iron metabolism is central to ferroptosis, a form of non-apoptotic regulated cell death (III) (362–364, 366).	High levels are associated with a higher cancer risk (mainly HCC^b^) (11). Iron metabolism is dysregulated in cancer: it acts as a tumor growth factor and is involved in DNA metabolism, cellular energy generation, and oxidative stress (III) (355–361). Ferroptosis may favor cancer development (III) (366)
Zinc	Antioxidant effect. Zinc deficiency might increase the risk of endometrial (III) (369, 370), prostate (III), pancreatic (III) (368), breast (III) (368), gynecological (III) (371), and lung cancer (I) (372).	Supplementation might increase the risk of prostate cancer (III) (373).
Copper	“Cuproptosis”: cytotoxicity by ROS accumulation and high energy metabolism (III) (374, 375). Low copper levels might increase the risk of endometrial (III) (369, 370), gynecological (III) (371), and lung cancer (I) (372).	“Cuproplasia”: increased copper levels evidenced in several cancer types (III) (374).
Selenium	Antioxidant effect, enhances immune surveillance of cancerous cells, alteration of tumor cell metabolism. Low selenium levels might increase the risk of thyroid cancer (I) (383), prostate, lung, and colon cancer (III) (381, 382). Supplementation may reduce toxicity from radiotherapy in gynecological cancer patients (387, 388).	Unknown.
Chromium	Unknown.	Cancerogenic for IARC^c^ (lung cancer) (I) (10, 354). Possible higher occupational risk of several other cancer types, in particular gastric cancer (I) (389, 390).
Iodine	Anti-proliferative and pro-apoptotic effect on breast and gastric cancer cells (III) (393).	Unknown.
Manganese	Antitumor immune response (III) (396).	Unknown.
Molybdenum	Anti-angiogenic agent (inducing copper deficiency) (III) (397). High levels associated with a lower risk of pancreatic cancer (III) (404). Essential cofactor for several enzymes that can have a central role in hindering oncogenesis (III) (398–401).	Essential cofactor for several enzymes that can have a central role in favoring oncogenesis (III) (399, 402, 403).

Level of evidence: I -> meta-analysis or randomized controlled trial, II -> prospective cohort, III -> retrospective cohort or preclinical evidence. Please see the text for accurate references for each finding.

^a^ROS, reactive oxygen species.

^b^HCC, hepatocellular carcinoma.

^c^IARC, International Agency for Research on Cancer.

Furthermore, aligned with the principles of culinary medicine (19), we included a paragraph aimed at integrating nutrition, medicine, and culinary arts while also addressing patients' frequent requests for nutritional counseling during oncologic treatment or follow-up. As a “proof-of-concept” section, we have developed a set of recipes for patients and caregivers to manage hypokalemia and hypocalcemia (which are particularly common in clinical practice), which may serve as useful tools for clinicians and dietitians.

## 2 Material and methods

The literature search was conducted using PubMed with specific keywords and Boolean operators. For each micronutrient considered, the search was performed by two different authors.

For each micronutrient, the following keywords were used:

- ((nutrient) OR (nutrient deficiency)) AND ((cancer prevention) OR (cancer protection))
- ((nutrient) OR (nutrient deficiency)) AND ((protective effect) AND ((cancer) OR (oncology)))
- ((nutrient) OR (nutrient deficiency)) AND ((cancer risk) OR (cancer incidence) OR (cancer development))
- ((nutrient) OR (nutrient deficiency)) AND (supplementation) AND ((cancer) OR (oncology))
- ((nutrient) OR (nutrient deficiency)) AND (((toxicity) OR (toxicities) OR (adverse events) OR (adverse effects)) AND ((cancer) OR (oncology)))

Only scientific articles (international guidelines, clinical trials, meta-analyses, systematic and narrative reviews) published until January 2025 and written in English were included. Additional references were added through cross-referencing or were already known.

## 3 Vitamins

Vitamins are essential organic compounds needed in small amounts to support normal metabolic functions and health (Table 1) (4). Unlike macronutrients such as carbohydrates, proteins, and fats, vitamins do not provide energy but are crucial for a wide array of biochemical processes, including cellular metabolism, immune function, and the maintenance of tissue integrity. They are classified into two main categories: water-soluble and fat-soluble. Water-soluble vitamins, like the B-complex group and vitamin C, must be consumed regularly, as they are not stored in the body. Fat-soluble vitamins, including vitamins A, D, E, and K, are stored in the liver and adipose tissues, allowing them to be utilized as needed.

In oncology, the role of vitamins is particularly important (Table 3) (5, 6). Cancer patients often face challenges such as inadequate nutrient intake, altered metabolism, and increased nutritional needs, which may lead to deficiencies (5). Proper assessment and management of vitamin status can enhance quality of life, support treatment efficacy, and potentially improve clinical outcomes.

In the following sections, we will provide an in-depth look at each vitamin and explore its specific role in cancer care.

### 3.1 Vitamin A

Vitamin A is a fat-soluble vitamin, and the term usually refers to all retinoids. Carotenoids are provitamins (i.e., precursors of vitamin A) produced by plants that can be converted into retinal by the human body. Vitamin A serves essential functions: it supports vision as a key component of rhodopsin, regulates keratinocyte and myeloid cell differentiation, modulates gene expression by binding to nuclear receptors [retinoic acid receptor (RAR) and retinoid X receptor (RXR)], and plays roles in bone development and immune function (4). It is absorbed in the small intestine, with a recommended daily intake of 900 μg for men and 700 μg for women (4). Retinoids are found in foods of animal origin, primarily meat, eggs, dairy products, and fish; carotenoids are present in significant amounts in yellow, orange, and red fruits and vegetables. The vitamin A content in foods can be reduced by oxidation (4).

Acute deficiency is rare in Western countries; people at higher risk generally suffer from malabsorption syndromes or have undergone bariatric surgery. Typical deficiency symptoms include fatigue, xerophthalmia, impaired night vision, dry skin and hair, a weakened immune system, and infertility (4, 20). Diagnosis is typically clinical but can be confirmed with retinol serum level measurement. Treatment of deficiency in adults consists of 200,000 IU given orally, starting from the diagnosis, with subsequent doses after 24 h and then after 2 weeks.

Cancer patients often have subclinical vitamin A deficiency. Patients who have undergone gastrectomy or pancreatectomy may have impaired absorption of all fat-soluble vitamins, including vitamin A (21).

Liver diseases, such as metabolic dysfunction-associated steatotic liver disease (MASLD), are linked to altered vitamin A homeostasis, but it remains unclear whether a vitamin A deficit contributes to the pathogenic mechanisms that can progress from MASLD to HCC (22). In animal models, vitamin A deficiency has been associated with cigarette smoke exposure, leading to an increased risk of lung cancer (23). The mechanisms by which vitamin A deficiency may promote lung cancer, potentially by altering the immune response, are still under investigation (24). However, supplementation with high doses of beta-carotene (averaging 20–30 mg daily in clinical trials) has also been linked to a higher risk of lung cancer in smokers or ex-smokers (6, 25). High vitamin A intake has been associated with a lower risk of gastric cancer and cutaneous squamous cell carcinoma (26, 27), while a potential association between vitamin A deficiency and a higher risk of cervical and colorectal cancer has been observed in animal models (28, 29). However, all clinical trials have failed to confirm a beneficial preventive effect of vitamin A supplementation; therefore, its clinical application remains limited.

Retinoids have also been tested in the past as therapeutic agents for many cancer types, due to their ability to promote differentiation and potentially induce cancer cell death. All-trans retinoic acid (ATRA), the active form of vitamin A, binds to nuclear retinoic acid receptors (RARα/β/γ), regulating various biological processes, such as cell proliferation, differentiation, and apoptosis (30). The expression of RARs is often dysregulated in several cancer types (31, 32). In addition to its genomic effects, ATRA can also act through non-canonical mechanisms, for example, by interacting with the aldehyde dehydrogenase (ALDH) enzyme family or modulating the immune response, potentially with a pro-tumorigenic role (31, 33–35). Several studies have been published on its role in acute promyelocytic leukemia, where it represented one of the first examples of targeted therapies and has been used as a chemotherapeutic drug for years (36). However, there is also considerable data for other solid cancers, suggesting a potential role in reducing cell proliferation and invasiveness, modulating gene expression, repairing DNA damage, and possibly sensitizing to systemic therapies (37–45).

### 3.2 Vitamin D

**Figure 1:**
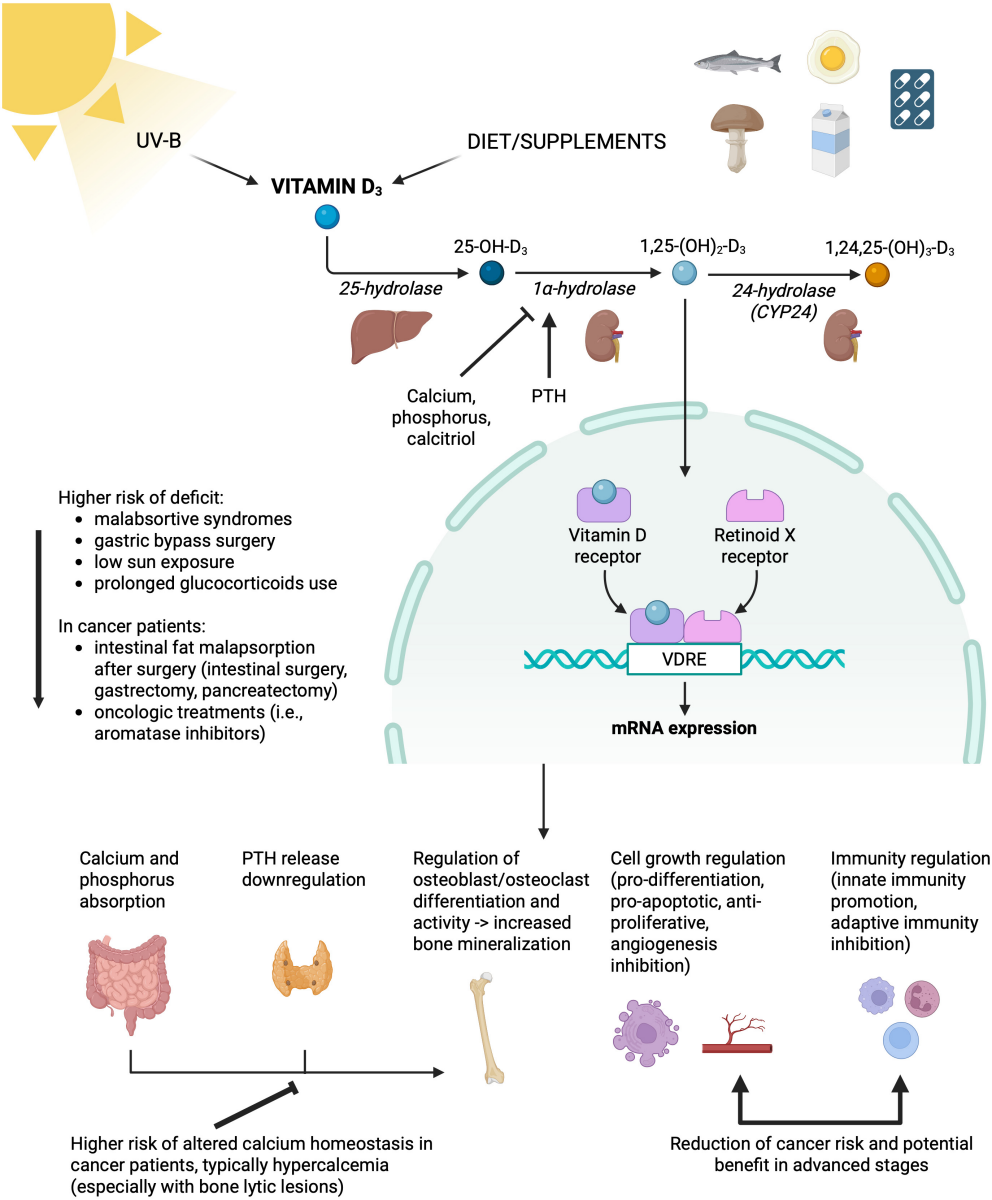
Vitamin D and calcium homeostasis and their potential role in cancer patients. PTH, parathyroid hormone; VDRE, vitamin D responsive element. Image created in BioRender by de Scordilli, M. in 2025. https://BioRender.com/hedgi1j.

Vitamin D, or calciferol, is a fat-soluble vitamin essential for calcium homeostasis and bone health. It can be produced by skin cells with exposure to ultraviolet sunlight, which represents the major natural source, as vitamin D intake from diet is limited. It regulates osteoblast and osteoclast activity for bone metabolism, promotes calcium and phosphate absorption, downregulates parathyroid hormone (PTH) release, and supports immune and muscle function (4) (Figure 1). Vitamin D is naturally present in very few foods (i.e., fatty fish and fish oil, with smaller quantities in mushrooms, egg yolks, and liver) but is commonly added to cereals and bread products. It is absorbed in the small intestine, and a daily intake of 600 IU (15 μg) is recommended for both men and women (800 IU (20 μg) for those over 70 years of age) (4).

Vitamin D deficiency can result from inadequate intake or absorption, or from hepatic and/or renal insufficiency, which impairs the conversion of the vitamin to its active form. Clinical manifestations of severe deficiency, such as rickets and osteomalacia, are now extremely rare; in contrast, subclinical deficiency is common and may present with hypophosphatemia, hypocalcemia, secondary hyperparathyroidism, bone demineralization, and an increased risk of fractures. A higher risk of deficiency is present in patients with malabsorptive syndrome and/or unusually low sun exposure, those who have undergone gastric bypass surgery, or those on prolonged glucocorticoid use (which alters intestinal absorption) (46). Daily oral doses of 800–2,000 IU (20–50 μg) for 2–3 months are recommended for adults with mild deficiency, while severe deficits are usually treated with higher doses (1,250 μg) once per week for 6–12 weeks, followed by a lower daily intake.

Cancer patients may have a higher risk of deficiency due to causes of intestinal fat malabsorption (i.e., intestinal surgery, pancreatectomy, gastrectomy) or due to oncologic treatments, such as aromatase inhibitors.

Vitamin D active ligand (1,25-dihydroxyvitamin D3 or calcitriol) normally binds to the VDR nuclear receptor, forming a heterodimer with the Retinoid X Receptor (RXR), which interacts with vitamin D response elements (VDREs) to regulate gene transcription (47). In this way, calcitriol may play a central role in several intracellular molecular pathways, including carcinogenesis. VDR functions as a tumor suppressor by antagonizing the Wnt/β-catenin pathway, thereby inhibiting cancer cell proliferation and invasiveness and promoting apoptosis in several tumor types [i.e., colorectal (48, 49), breast (50), gastric cancer (51), melanoma (52)]. The antiproliferative effect of calcitriol involves multiple pathways, such as the expression of cyclin-dependent kinase inhibitors (i.e., CDKN1A and CDKN1B), the production of growth factors (i.e., TGF-β, IGF) and miRNAs, the regulation of cancer stem cell proliferation, tumor angiogenesis, epithelial-mesenchymal transition, and the activation of caspases (49, 53–56). High tumor VDR expression has also been associated with an upregulation of antitumor immunity (49, 52). Furthermore, vitamin D has shown potential in reversing drug resistance in several tumors in preclinical studies (55).

It has been shown that high levels of vitamin D are associated with a lower risk of many cancer types (57–63). However, in most clinical trials, the potential benefit of vitamin D intake for cancer prevention has not been confirmed (64–66). In a recent trial, a significant reduction in advanced cancers was observed in patients randomized to vitamin D supplementation (67); moreover, a meta-analysis of previous randomized trials showed a reduction in total cancer mortality with supplementation (68). Additionally, a deficiency of vitamin D has been associated with worse adverse effects during oncologic treatments (69, 70).

A possible role of vitamin D supplementation in promoting cancer progression has also been reported, although the evidence is limited and primarily derived from observational associations. In a nested case–control study involving prostate cancer patients and matched controls, both low (≤19 nmol/L) and high (≥80 nmol/L) serum levels were associated with a higher risk of prostate cancer, while average concentrations were linked to the lowest risk (71). A meta-analysis of prospective studies confirmed a significant correlation between higher (vs. lower) vitamin D concentrations and an increased risk of prostate cancer (RR 1.15; RR 1.04 for a 10 ng/mL increment) (72). In cancer patients, a detrimental effect of vitamin D supplementation may be associated with defective VDR signaling, according to data from preclinical studies: VDR deficiency could lead to the loss of vitamin D's beneficial effects and possibly the emergence of harmful effects, such as the upregulation of tumor angiogenesis and suppression of anti-tumor immunity (73–77).

### 3.3 Vitamin E

Vitamin E, a fat-soluble vitamin primarily in the form of alpha-tocopherol, functions as an antioxidant, protecting cell membrane integrity. It also supports immune health and may help prevent atherosclerosis and cardiovascular diseases (4). Vitamin E cannot be produced by the human body, and its absorption occurs in the small intestine, with a recommended daily intake of 15 mg (4). It is found in plant oils, nuts, seeds, fruits, and vegetables.

Vitamin E deficiency is rare; it is mainly due to malabsorptive syndromes (i.e., previous GI surgery, cholestatic liver disease with low bile concentration in the intestine) and clinically presents with altered immune function, retinopathy, peripheral neuropathy, ataxia, and hemolysis (21, 78). Vitamin E serum levels can be assessed to confirm deficiency and must be monitored in patients at high risk of insufficiency. Oral supplementation is usually sufficient to treat deficiencies, with 17–35 mg/kg per day of alpha-tocopherol. In cases of chronic malabsorption, replacement therapy is needed with variable doses (50–500 mg/day according to serum concentration) (78).

There is no clear evidence that vitamin E supplementation has a beneficial effect in reducing cancer incidence (12, 79–83); a single prospective study demonstrated an association between long-term supplementation of alpha-tocopherol and lower incidence and mortality of prostate cancer in male smokers (84). In contrast, a mild increase in the risk of prostate cancer with high vitamin E supplementation has been reported in a randomized trial (85). Several studies are still ongoing, including those involving other vitamin E isoforms (86).

Also, a possible association between dietary vitamin E and increased immunotherapy efficacy has been recently reported, even though in preclinical models (87). The impact of supplemental intakes on the response to systemic therapies remains unexplored. Preclinical studies have shown the anti-tumor activity of δ-tocotrienol on pancreatic cancer cells, with the suppression of NF-κB activation and induced apoptosis (88–90). The concomitant administration of δ-tocotrienol has demonstrated an increased efficacy of gemcitabine (88), and a phase I study on pancreatic cancer patients was conducted with escalating doses of δ-tocotrienol in the 2 weeks before surgery (91).

### 3.4 Vitamin K

Vitamin K is a fat-soluble vitamin that plays fundamental roles linked to the coagulation pathway and the activity of several protein enzymes. It is also involved in bone mineralization and the prevention of vascular calcification and coronary heart disease (4, 92). Vitamin K cannot be produced physiologically by human cells but can be synthesized by the gut microbiota (vitamin K2 or menaquinone) (4). Vitamin K1 (phylloquinone) from dietary intake is absorbed in the small bowel; the recommended daily intake is 120 μg for men and 90 μg for women, and it is mainly found in green vegetables (4). Vitamin K2 can also be found in animal foods (4).

Vitamin K deficiency is rare in adults, primarily due to malabsorption, total parenteral nutrition, or antibiotic use (which reduces gut bacteria production of vitamin K), a common issue in oncology wards. Clinical presentation can include melena or hematuria, mucosal bleeding, skin bruises, and osteopenia/osteoporosis. Deficiency can be diagnosed by evaluating prothrombin time (PT) and the international normalized ratio (INR), which are usually prolonged. An indirect evaluation can be made by measuring the levels of vitamin K-dependent coagulation factors, which are below 50% in the presence of vitamin deficiency. Coagulopathy can be quickly treated with a single dose of 10 mg (to be administered parenterally in patients with impaired absorption), which can be repeated after 48–72 h (4, 92).

No clear data are available in the literature regarding the association of vitamin K intake with cancer incidence (93, 94). Menaquinone intake has been linked to an increased risk of breast cancer (95), while no association has been shown between the dietary intake of phylloquinone, menaquinone, or total vitamin K and the risk of prostate cancer (96). A prospective study has demonstrated an association of phylloquinone and dihydrophylloquinone (but not menaquinone) intake with a lower risk of pancreatic cancer (97). Another prospective cohort study indicated an overall lower cancer incidence and mortality with higher dietary intake of menaquinone (98). Moreover, vitamin K1 intake has been associated with a reduction in cancer-related mortality in current/former smokers (99), and vitamin K consumption has been linked to a lower incidence of lung cancer (100). Based on this data, interventional randomized trials are still awaited.

Vitamin K3 (menadione) has demonstrated the ability to suppress epithelial–mesenchymal transition and the Wnt signaling pathway in colorectal cancer cells, decreasing cell proliferation, invasion, and migration (101). Menaquinone has shown anticancer activity in leukemia cells, inducing autophagy and apoptosis (102, 103). Additionally, menaquinone can induce both apoptotic and non-apoptotic cell death in cancer cells in solid tumors (104–106). An inhibitory role of vitamin K in HCC development has been reported in preclinical models (107, 108), and it is currently being studied as a potential cancer chemosensitizer in combination with other oncologic drugs (109). Vitamin K2 intake has been studied in curatively resected HCC patients but has not shown efficacy in reducing the risk of recurrence or death in this setting (110).

### 3.5 Vitamin C

Vitamin C, or ascorbic acid, is a water-soluble vitamin that modulates immune function, supports collagen synthesis, and aids in wound healing. As a reversible electron donor, it acts as an antioxidant and cofactor in various enzymatic processes (111–114). Ascorbic acid cannot be synthesized by the human body and is absorbed in the distal small intestine. A daily intake of 90 mg for men and 75 mg for women is recommended, with an additional 35 mg intake recommended for smokers (4). The best food sources include fruits and vegetables, mainly citrus, strawberries, tomatoes, cruciferous vegetables, and potatoes (4). High-heat cooking and oxidative conditions can deplete the vitamin content of foods.

Vitamin C deficiency is rare in developed countries; higher-risk conditions include diets restricted in fruits and vegetables, smoking, and drug and alcohol abuse. Scurvy is the clinical manifestation of severe deficiency, with signs and symptoms linked to a weakening of connective tissues. Symptoms of mild deficiency include fatigue, arthralgias, and anemia due to decreased absorption of non-heme iron (115). The diagnosis of deficiency is usually clinical, and it is treated with oral, intramuscular, or intravenous supplementation, typically with 300–1,000 mg/day for 1 month.

The role of vitamin C in cancer patients has been studied for years. Cancer patients may experience ascorbic acid deficiency due to reduced intake or altered absorption, systemic inflammation, or treatment consequences (116).

Considering its antioxidant effect in neutralizing reactive oxygen species (ROS), vitamin C may play a role in preventing DNA damage and cancer transformation (117, 118). High plasma levels of vitamin C have been associated with a lower risk of gastric cancer (119, 120) and overall cancer of the digestive tract (121), possibly linked to its neutralizing effect on N-nitroso compounds derived from red and processed meats. A correlation between vitamin C intake and a reduced incidence of pancreatic cancer has also been described (122). However, randomized trials on its potential use for cancer prevention have mostly yielded negative results (12). Ascorbic acid has also been studied in the therapeutic setting for cancer patients, considering that high doses act as pro-oxidants rather than antioxidants and can limit the glycolysis-dependent energy production of cancer cells by hindering transporter-mediated glucose intake (117, 123). Therapeutic doses of vitamin C may act against cancer development and progression through several other mechanisms, such as stimulating immune system activation or enhancing the chemosensitivity of cancer cells, but evidence beyond preclinical models is still lacking (124, 125). Oral intake alone does not provide sufficient absorption to reach therapeutic doses, with plasma concentration always < 250 mmol/L, while intravenous administration can lead to plasma levels 30–70 times higher than the highest tolerated oral dose (126). Nevertheless, even clinical trials investigating high-dose intravenously administered vitamin C, both alone and in combination with standard chemotherapy (CT), have not shown positive outcomes in terms of antitumor efficacy, increased CT effectiveness, or reduced CT-induced toxicities (124).

### 3.6 Vitamin B1 (thiamine)

Vitamin B1, or thiamine, is a water-soluble vitamin essential as a coenzyme in the conversion of pyruvate to acetyl-coenzyme A, a key step in the Krebs cycle. Additionally, it plays a role in nerve impulse transmission by modulating membrane permeability, neurotransmitter production, and myelin sheath maintenance (4, 127). Thiamine cannot be produced by the human body and is absorbed in the small intestine, with a recommended daily intake of 1.2 mg for men and 1.1 mg for women (4). Foods rich in thiamine include legumes, meat, fish, cereals made from whole grains or thiamine-enriched (though it is very low in processed cereals), yeast, brown rice, and milk (4).

Thiamine deficiency primarily affects tissues that require a constant supply of energy, with symptoms including congestive heart failure, muscle weakness, and neurological manifestations (128). Deficiency can be linked to low intake of thiamine-rich foods, malabsorption, refeeding syndrome in patients with severe malnutrition, or increased urinary loss due to diuretics or alcohol abuse (which impairs thiamine absorption in the intestine). A typical clinical presentation is Wernicke–Korsakoff syndrome, common in cases of alcohol abuse, characterized by an acute confusional state and ophthalmoplegia (129). Another notable clinical condition resulting from severe thiamine deficiency is beriberi, which presents with a wide range of symptoms (130). Thiamine concentration can be directly measured in the blood; however, levels may be falsely reduced due to hypoalbuminemia, systemic inflammation, or in critically ill patients (115, 131). Empiric therapy should be considered with a high suspicion of thiamine deficiency, using high-dose oral supplements or intravenous supplementation, along with a balanced diet.

Cancer patients can experience thiamine deficiency due to multiple factors, such as malabsorption possibly resulting from surgery, a systemic inflammatory state, accelerated thiamine usage by tumor cells, malnutrition, and CT administration. Conditions considered particularly high risk include gastrointestinal (GI) and hematological cancers, as well as patients undergoing chemotherapy (132–135), advanced age, significant weight loss, and hypoalbuminemia.

B vitamins play an important role in immune cell regulation; however, although the immune system is a key factor in cancer suppression, the beneficial or detrimental role of B vitamins in cancer development has not yet been fully clarified (13). Hypotheses involving thiamine relate to its possible role in mediating the metabolic processes associated with increased proliferation and energy demands of tumor cells. In particular, thiamine appears to have a dual role: a slight increase in its concentration may enhance tumor growth via thiamine-dependent enzymes (i.e., transketolase, pyruvate dehydrogenase), while high doses may shift tumor metabolism toward oxidative pathways, reducing glycolysis (and the Warburg effect) (136–138). Several thiamine homeostasis genes exhibit altered expression in solid cancers in preclinical models, particularly concerning thiamine transporters (i.e., upregulation of the gene expression of TPK1, *SLC19A2*, and *SLC25A19*, and downregulation of *SLC19A3*), resulting in high levels of intracellular free thiamine (137). The alterations in thiamine homeostasis and the increased thiamine-dependent cell proliferation underscore its significant role in cancer (137). However, high doses can reduce proliferation in cancer cell lines (137, 139). Thiamine also appears to have a protective effect on colorectal cancer (140); a protective effect has also been reported for bladder cancer (141) and hormone receptor (HR) negative/human epidermal growth factor receptor 2 (HER2) positive breast cancer (142). Data from interventional randomized trials are still lacking, however.

### 3.7 Vitamin B2 (riboflavin)

Vitamin B2, also known as riboflavin, is a water-soluble vitamin of the B complex involved in redox reactions of many crucial metabolic pathways through the biosynthesis of the flavocoenzymes flavin mononucleotide (FMN) and flavin adenine dinucleotide (FAD). It also contributes to the regeneration of glutathione, providing antioxidant effects (143). Moreover, recent evidence suggests its role in fetal development, hematological status, and neuroprotection (144). Riboflavin cannot be synthesized by the human body; it is absorbed in the intestine, with a recommended daily intake of 0.9–1.1 mg for women and 1.1–1.3 mg for men (145). It is present in a wide variety of foods, such as milk and dairy products, meat, and green vegetables (145). Notably, food processing can alter vitamin bioavailability, impairing the nutritional quality of foods (146).

Symptoms related to vitamin B2 deficiency are non-specific, as it is usually concomitant with other vitamin B deficiencies. Overall, symptoms include fatigue, swollen throat, ocular manifestations such as blurred vision (147), and depression (148, 149); patients may also present mucocutaneous lesions (i.e., dermatitis, itching, and glossitis) (150). Following clinical suspicion, riboflavin deficiency can be confirmed by measuring its urinary excretion (151). Supplementation consists of oral or intramuscular riboflavin, with doses typically ranging from 5 to 10 mg per day; however, treatment should be personalized, considering gender, age, causes, and severity of insufficiency (151).

Cancer patients may have predisposing conditions that increase the risk of riboflavin deficiency, such as diarrhea and vomiting due to cancer treatments, intestinal or gastric resection for primary GI tumors, reduced intake, and cachexia (152, 153). Decreased blood levels may be associated with defective gene expression for transporters (such as *RFT2* in gastric cancer and C20orf54 gene in esophageal squamous cell carcinoma) (154, 155).

Limited and mostly preclinical evidence is currently available regarding the interplay between riboflavin and cancer. In preclinical models, riboflavin deficiency decreases glutathione reductase activity, causing oxidative damage and altering DNA repair enzymes, suggesting a pro-carcinogenic environment at low levels (156, 157). However, this vitamin seems to play a role in cancer development and progression, as the proteins responsible for its transport are overexpressed in several tumor types (158–160). Observational studies have described an association between high serum riboflavin and a higher risk of pancreatic and colorectal cancer (161, 162). The specific molecular mechanisms involved in the deregulation of riboflavin transporter expression and their oncogenic roles are not yet fully elucidated (163). In esophageal squamous cell carcinoma (ESCC), diffuse cytoplasmic expression of *SLC52A3* (which codes for riboflavin transporter-3) can be observed in tumoral tissue compared to normal cells; additionally, in a cohort of ESCC patients, the nuclear expression of *SLC52A3* was associated with poor prognosis (164). These preliminary data suggest a possible prognostic and predictive role of riboflavin transport proteins, as well as a potential targetable pathway for tumor-targeted drug internalization (165, 166). Preclinical studies have also focused on the role of riboflavin as an endogenous photosensitizer, capable of generating ROS under irradiation, and therefore with possible applications in photodynamic therapy (167, 168).

### 3.8 Vitamin B3 (niacin)

Vitamin B3, also known as niacin or nicotinic acid, is an essential nutrient and part of the vitamin B complex. Its derivative form, nicotinamide, constitutes two cofactors with a pivotal role in oxidation–reduction reactions and in the modulation of several biological processes: nicotinamide adenine dinucleotide (NAD) and nicotinamide adenine dinucleotide phosphate (NADP+) (169, 170). Both niacin and niacinamide are absorbed in the stomach and small intestine, and the recommended daily intake in the European Union is 1.3 mg NE/MJ for females and 1.6 mg NE/MJ for males (171–173). Dietary sources include fish, meat, and legumes (174).

Severe deficiency is rare in Western countries and limited to at-risk groups (i.e., chronic alcoholism). It causes a disease called pellagra, characterized by diarrhea, photosensitive rash, stomatitis, glossitis, and both GI and central nervous system symptoms (175). The most reliable method to determine vitamin B3 levels is a 24-hour urine collection (176). Treatment for niacin deficiency includes a balanced diet, avoiding alcohol consumption, and supplementation with nicotinamide at doses of 250–500 mg/day (177).

In patients with carcinoid tumors, carcinoid syndrome can mimic pellagra-like manifestations and is likely due to the diversion of tryptophan from vitamin B3 synthesis to serotonin production (178). Other predisposing factors include intestinal malabsorption due to severe chronic diarrhea and concomitant medications such as isoniazid (179).

Niacin is indirectly involved through the NAD coenzyme in the regulation of DNA repair, genomic stability, and gene expression modulation, influencing cell survival and proliferation, a well-known hallmark of cancer (180). Preclinical evidence suggests that the disruption of these processes has the potential to deregulate cell division, increasing the risk of malignant degeneration (181). Therefore, nicotinic acid has been tested as a potential chemopreventive agent, especially for skin cancer, with controversial results: prospectively collected data showed an association between niacin intake and a reduced risk of squamous cell carcinoma, as well as a slightly increased risk of basal cell carcinoma and melanoma in men (182, 183). Randomized studies are certainly needed to clarify these data. Additionally, a systematic review indicated that vitamin B3 supplementation could be associated with a lower risk of hepatocellular carcinoma (14).

### 3.9 Vitamin B5 (pantothenic acid)

Vitamin B5 is an essential nutrient and a key precursor for the biosynthesis of coenzyme A (CoA), which plays a central role in hundreds of biochemical reactions (184). This vitamin cannot be produced physiologically and is predominantly absorbed in the jejunum; the recommended intake for adult men and women is 5 mg per day (173, 185). It is available from a wide variety of dietary sources (i.e., eggs, milk, meat, and vegetables) and is also produced by colonic bacteria (186).

As is often the case with vitamin deficiencies, clinical diagnosis is complex due to the coexistence of various deficits. Patients usually report fatigue, malaise, weakness, numbness, concentration difficulties, paresthesia, muscle cramps, and GI symptoms (187). Measurement of urinary excretion is currently considered the most consistent method to determine vitamin B5 deficiency (188). Dietary supplementation should not exceed 10 mg per day (189).

Cancer patients with chronic severe malnutrition could be at risk for vitamin B5 deficiency, as could patients with malabsorption due to jejunum resection for primary intestinal tumors (190).

Currently, no solid evidence is available regarding its possible role in carcinogenic processes (13). In preclinical models, high vitamin B5 concentration has been associated with breast cancer cells exhibiting higher metabolic activity through the c-MYC oncogenic pathway: the overexpression of the multivitamin transporter SLC5A6 has been described, leading to increased conversion to CoA and cell proliferation (191).

Conversely, *in vitro* administration of CoA or pantethine (a vitamin B5 precursor) can increase CD8+ T cell anti-tumor activity (192, 193); pantethine administration has shown an effect in reducing tumor growth and metabolism in ovarian cancer cells (194). Also, vitamin B5 supplementation can enhance the efficacy of anti-PD-L1 therapy in animal models (192). Higher B5 plasma levels have been associated with responses to immune checkpoint inhibitors in a small cohort of melanoma patients, suggesting that more studies are needed to confirm the possible role of B5 in anti-cancer immunosurveillance (192, 195).

### 3.10 Vitamin B6 (pyridoxine)

Vitamin B6 is a water-soluble compound; its active form is the coenzyme pyridoxal 5-phosphate (PLP or P5P), which is involved in many biochemical processes, including gluconeogenesis, glycogenolysis, and amino acid, lipid, and carbohydrate metabolism (196, 197). It cannot be produced by the human body and has intestinal absorption; the recommended dietary allowance depends on age: 1.3 mg per day for young males and females, and 1.7 mg and 1.5 mg per day for men and women older than 50 years, respectively (171, 173). It is commonly present in plant foods.

The most frequent clinical presentations of vitamin B6 deficiency include skin and mucosal manifestations, neurologic impairment, and a decrease in red and white blood cell counts (198–200). Several laboratory tests are available to detect vitamin B6 levels in plasma and urine (201). Oral supplementation in cases of deficiency should not exceed 50 mg/day and should be administered for less than 6 months (202).

Risk factors for vitamin B6 deficiency in cancer patients include severe malnutrition or malabsorption, parenteral nutrition, and concomitant medications that interfere with pyridoxine.

Current evidence exploring the potential relationship between vitamin B6 and cancer represents a promising research field. A recent meta-analysis has shown that blood PLP levels are inversely associated with the risk of developing colorectal cancer; however, no direct association has been found between vitamin B intake and colorectal cancer risk (203). Vitamin B6 supplementation could be associated with a lower risk of nasopharyngeal carcinoma, pancreatic cancer, and breast cancer (14). Additionally, low vitamin B6 levels could increase cancer risk by favoring DNA aberrations, as one of its roles is to transfer 1-carbon groups for DNA synthesis and methylation (203). Finally, in a small number of patients, vitamin B6 and B12 supplementation combined with acupuncture was effective as adjunct therapies in reducing chemotherapy-induced peripheral neuropathy (14). A randomized study may be needed to confirm these observations.

### 3.11 Vitamin B7 (biotin)

Biotin is a water-soluble vitamin that acts as a coenzyme in several biological processes, including gluconeogenesis, fatty acid synthesis, and amino acid metabolism (204). Biotin is mainly found in meat and eggs but can also be synthesized by gut bacteria (205); absorption is non-saturable and occurs in the proximal small intestine and in the cecum (171). The recommended daily intake is 25 μg/day for those aged 14–18 years, and 30 μg/day for those 19 years or older (173).

Clinical presentations of biotin deficiency include dermatological abnormalities, neurological symptoms, GI manifestations, and depression (206, 207). The most reliable indicator for detecting deficiency is measuring the urinary excretion of 3-hydroxyisovaleric acid (208). Treatment for deficiency is based on oral supplementation with a minimum dose of 5 mg/day (209).

Cancer patients who require total parenteral nutrition are at increased risk of biotin deficiency and require supplementation (210). As with other vitamin depletions, a severe state of malnutrition or malabsorption can affect biotin uptake, as well as concomitant treatment with carbamazepine, phenytoin, and phenobarbital (211).

No current evidence is available regarding a possible role of biotin in carcinogenesis or cancer prevention.

### 3.12 Vitamin B9 (folate)

Folate is an essential water-soluble vitamin; its active form (i.e., tetrahydrofolate, THF) plays a crucial role in DNA synthesis, repair, and methylation through the biosynthesis of both purines and pyrimidines (171, 212). Folate is absorbed in the jejunum and is then converted to its active form by dihydrofolate reductase in the liver. The recommended intake for adults older than 14 years is 400 μg/day, and it is primarily found in fruits, green leafy vegetables, and liver (173).

Folate deficiency should be suspected in cases of megaloblastic anemia; a differential diagnosis with cobalamin deficiency should be carried out by measuring serum folate concentrations (213). Folate deficiency manifests as fatigue, muscle weakness, infertility, increased risk of cardiovascular disease, neurological manifestations, depression, mouth sores or ulcers, and symptoms related to macrocytic anemia (214, 215). The synthesized form of folate, folinic acid, is used as an oral supplement (1–5 mg daily) in cases of deficiency, as it is characterized by elevated bioavailability (213).

In cancer patients, folate depletion can occur as a consequence of malnutrition or malabsorption, but also as an adverse effect of oncological treatment with the antifolate drug methotrexate (216).

Chronic folate insufficiency has been linked to an increased risk of several solid tumors in various preclinical and epidemiological studies (217–219). Conversely, clinical trials demonstrated that folate supplementation can promote malignant degeneration of preneoplastic colorectal lesions and does not reduce the risk of colorectal adenomas (15, 220). The relationship between folate status and carcinogenesis is extremely complex. Some authors speculate that folate supplementation could exert its oncoprotective action by enhancing genomic stability, while aberrant DNA methylation in folate deficiency could increase cancer risk, suggesting a dual modulatory effect (221–223). Further interventional trials are needed to investigate the possible increased risk of colorectal cancer associated with folate supplementation.

### 3.13 Vitamin B12 (cyanocobalamin)

Vitamin B12, also known as cobalamin or cyanocobalamin, is an organic compound essential for DNA synthesis, fatty acid and amino acid metabolism, nervous system development and function, and red blood cell formation (224). It cannot be produced by the human body and is absorbed through two mechanisms: the first is more complex and involves the intrinsic factor (IF), while the second is mediated by passive diffusion (171, 225). Dietary recommendations vary from the USA to European countries: the US National Academy of Medicine recommends a dietary allowance of 2.4 μg/day for people aged 14 years or older, while the European Food Safety Authority suggests higher intakes, namely 4.0 μg/day for adults (226, 227). It may be found in dairy products, eggs, fish, and meat.

Vitamin B12 deficiency is associated with a broad spectrum of symptoms: fatigue, weakness, dizziness, neurological deterioration, glossitis, weight loss, infertility, megaloblastic anemia, and decreased platelet and white cell counts. Elderly patients can also experience psychiatric manifestations (228). B12 deficiency should be suspected in cases of megaloblastic anemia and confirmed by measuring B12 serum levels (229). Vitamin B12 can be supplemented in the form of cyanocobalamin or hydroxocobalamin, either orally or by parenteral route (230).

Cancer patients who have undergone total or partial gastric or intestinal resection should be screened for cobalamin deficiency (231–234). Moreover, chronic malnutrition, malabsorption, and pancreatic insufficiency represent additional risk factors (235–237). Clinicians should be aware that chronic treatment with proton pump inhibitors (PPIs) may reduce vitamin B12 absorption (238); therefore, therapy should be administered for a limited timeframe.

Evidence regarding a link between vitamin B12 and cancer is controversial. Some observational studies found an increased risk of cancer in subjects with elevated serum B12 levels compared to normal levels, suggesting a potential involvement of B12 in carcinogenesis (16, 239–241). A retrospective study on B12 plasma measurement in patients diagnosed with solid cancer showed a significant decrease in cases that were curatively treated (242). However, folate and vitamin B12 supplementation has been shown to reduce carcinogen-induced oxidative stress in colon cancer cells in animal models (243). A prospective observational study has indicated a lower risk of colorectal cancer with higher vitamin B12 plasma concentrations (244). Additionally, in colorectal cancer patients, B12 serum levels are inversely associated with DNA methylation, suggesting its potential role in the epigenetic regulation of several cancer pathways (245). Conversely, folic acid and vitamin B12 supplementation have been associated with an increased cancer risk (246–248).

As mentioned previously, in a small number of patients, vitamin B6 and B12 supplementation with acupuncture was effective as adjunct therapies in reducing chemotherapy-induced peripheral neuropathy (14). Folic acid and vitamin B12 are commonly administered to patients undergoing antifolate chemotherapy (i.e., pemetrexed) to reduce the incidence of high-grade hematologic and gastrointestinal toxicities (249).

### 3.14 Choline

Choline is a water-soluble nutrient and an acetylcholine precursor that is metabolized in the liver into major components of cell membranes (4). However, endogenous production is not sufficient to meet physiological needs. It is a key factor in neurotransmission, homocysteine metabolism, and many other metabolic processes; choline also plays a protective role in the cardiovascular system and is associated with lower concentrations of systemic inflammatory markers (250). Nevertheless, excessive choline intake and high levels of phosphatidylcholine have been linked with a higher risk of cardiovascular disease and type 2 diabetes mellitus (251, 252). Moreover, sphingomyelin plays a key role in the protective coating of nerves and in nerve impulse transmission. It is an essential nutrient absorbed in the small bowel, with a recommended daily intake of 550 mg in men and 425 mg in women (4). Foods rich in choline include egg yolks, beef liver, salmon, and legumes like soybeans and peanuts (4).

Choline deficiency rarely leads to clinically apparent deficits (115). Low choline levels are associated with accelerated atherosclerosis, a higher risk of cardiovascular disease, muscle damage, and MASLD (253, 254), as well as neurologic disorders (255). There is no definitive clinical test to identify choline deficiency. A diet that includes choline-rich foods is usually sufficient to avoid deficiency; choline is also available in dietary supplements in various forms, either by itself or in combination with B-complex vitamins.

Choline is not typically added to solutions used for parenteral nutrition, so cancer patients receiving total parenteral nutrition are at higher risk of developing choline deficiency and MASLD (256–258). Patients with intestinal failure resulting from surgery may also be at higher risk due to malabsorption (259).

Choline deficiency has been associated with a higher risk of HCC, through both direct and indirect mechanisms. Firstly, MASLD and metabolic dysfunction-associated steatohepatitis (MASH) are known risk factors for HCC. Moreover, other mechanisms of carcinogenesis have been suggested, such as cell dysregulation due to improper DNA methylation and epigenetic modifications, DNA damage from oxidative stress induced by mitochondrial dysfunction, and increased sensitivity to chemical insults (260, 261). Dietary choline supplementation has shown promising results in MASLD-related HCC in animal models (262), but prospective studies are still lacking in the clinical setting. High serum levels of choline and altered microbiome choline metabolism have also been associated with a higher risk of colorectal cancer (263, 264). However, these data have not yet had a clinical impact, which still requires investigation.

## 4 Minerals

Minerals are inorganic elements that play a critical role in a wide array of physiological processes essential for maintaining health and preventing diseases (Table 2) (4). Unlike vitamins, which are organic compounds, minerals are derived from the earth and cannot be synthesized by living organisms. They are categorized into two groups based on the quantities required by the body: macrominerals, which include calcium, phosphorus, magnesium, sodium, potassium, and chloride, needed in larger amounts; and trace minerals, such as iron, zinc, copper, selenium, chromium, iodine, manganese, and molybdenum, which are required in smaller quantities.

In the following paragraphs, we will cover in detail each mineral and its putative role in cancer (Table 4).

### 4.1 Calcium

Calcium homeostasis is regulated by PTH and 1,25-dihydroxyvitamin D. PTH and other hormones manage bone remodeling by regulating the activity of osteoblasts and osteoclasts. Calcium plays a role in muscle contraction, nerve impulse transmission, intercellular communication, and membrane permeability regulation (4). Calcium absorption mainly occurs in the duodenum and jejunum. The recommended daily intake is 1,000 mg for adults, 1,200 mg for females aged 51 years or older, and for males aged 71 years or older (4). Calcium-rich foods include dairy products, dark green vegetables, legumes, certain soy products, fish, and nuts (4, 265).

Patients with severe hypocalcemia (below 7 mg/dL) present with increased neuromuscular excitability. The most typical signs of hypocalcemia are Chvostek's sign (facial muscle spasm elicited by tapping the parotid gland over the facial nerve) and Trousseau's sign (carpopedal spasm elicited by inflation of a blood pressure cuff). Severe cases of hypocalcemia may progress to tetany, seizures, or cardiac arrhythmias (266). Serum albumin, magnesium, free calcium, vitamin D, and PTH levels, as well as kidney function, must be assessed to understand the cause of hypocalcemia (267, 268). The treatment of hypocalcemia depends on its severity: for mild, asymptomatic, or chronic hypocalcemia, oral supplementation is the best option, while intravenous infusion is indicated for patients with severe symptomatic hypocalcemia (268).

Patients with malignancies more frequently experience hypercalcemia, especially those with bone lytic lesions (269). Hypocalcemia is rare and often associated with renal impairment, and it can also occur during bisphosphonate or denosumab treatment for bone metastases (270–272). Low albumin concentration, which is common in cancer patients, can cause the phenomenon of pseudo-hypocalcemia. Vitamin D deficiency can lead to an increase in PTH as a compensatory mechanism for hypocalcemia. Additionally, hypomagnesemia induces PTH resistance, resulting in hypocalcemia. Furthermore, bowel surgery or neck surgery/radiation can cause hypocalcemia due to calcium malabsorption or parathyroid dysfunction (273).

High daily intake of calcium (more than 700 mg per day) has been associated with a reduced risk of recurrence of colorectal polyps (274, 275) and of developing colorectal cancer (276–279). Calcium plays a critical role in intracellular signaling, particularly in regulating cell proliferation, differentiation, and apoptosis, all of which are tightly controlled processes that, when dysregulated, can lead to carcinogenesis (280). In the colon, high luminal calcium concentrations have been shown to induce differentiation of colonic epithelial cells while suppressing excessive proliferation (281). This is mediated through the activation of the calcium-sensing receptor (CaSR), which acts as a tumor suppressor in several epithelial tissues. CaSR activation can inhibit the Wnt/β-catenin pathway, a major oncogenic driver in colorectal cancer. It also modulates E-cadherin expression, enhancing cell–cell adhesion and reducing invasiveness. Additionally, calcium can trigger apoptosis via mitochondrial pathways. Elevated intracellular Ca^2^? levels can lead to mitochondrial permeability transition pore (mPTP) opening, cytochrome C release, and activation of the caspase cascade (282, 283). This selective pro-apoptotic effect may help eliminate precancerous or dysplastic cells from epithelial surfaces.

High intakes of calcium and vitamin D may also be associated with a lower risk of developing premenopausal breast cancer (9, 284).

All the evidence on the oncoprotective role of calcium intake comes from prospective cohort studies; therefore, randomized trials are still awaited.

### 4.2 Phosphorous

Plasma phosphorus concentration is regulated by fibroblast growth factor 23 (FGF23), PTH, and calcitriol. Together with calcium and magnesium, it is involved in bone mineralization by constituting hydroxyapatite. Moreover, phosphorus is a component of nucleic acids (DNA and RNA) and is fundamental for energy production through ATP (adenosine triphosphate). It also plays an important role in cell signaling and metabolism through phosphorylation/dephosphorylation processes. Additionally, phosphorus controls blood pH balance and constitutes phospholipid cell membranes (4). Phosphorus absorption occurs in the small intestine. The recommended daily intake for adults is about 700 mg (4). Phosphate-rich foods include dairy products, salmon, beef, legumes, nuts, seeds, and vegetables like asparagus, tomatoes, and cauliflower (4).

Phosphorus deficiency can be asymptomatic when its concentration ranges from 2 to 2.5 mg/dL, while severe deficiency occurs with levels under 1.5 mg/dL. A typical symptom of severe hypophosphatemia is muscle weakness due to rhabdomyolysis, which can also impact cardiac function, leading to fatal arrhythmias. Other possible clinical presentations include confusion, anorexia, inadequate bone mineralization leading to rickets/osteomalacia, neurological problems like ataxia and paresthesia, and rigidity of red blood cells resulting in hemolysis and anemia (285). Patients with asymptomatic or mildly symptomatic hypophosphatemia (1–1.9 mg/dL) can receive oral potassium/sodium phosphate tablets; those with severe symptomatic hypophosphatemia or who are unable to take oral supplementation can be treated with intravenous phosphate (285).

Patients diagnosed with cancer can experience hypophosphatemia because of malnutrition and anorexia, secondary to CT or the neoplastic site of growth (for example, GI tumors). Medications such as aluminum or magnesium antacids, or clinical conditions like chronic diarrhea, can cause phosphate malabsorption. Refeeding syndrome, which often occurs in cancer patients after a phase of malnutrition, is another cause of hypophosphatemia. Furthermore, several anticancer treatments can cause hypophosphatemia, including ifosfamide, cisplatin, and tyrosine kinase inhibitors (TKI) such as imatinib, sunitinib, sorafenib, and regorafenib, as well as anaplastic lymphoma kinase (ALK) inhibitors and mammalian target of rapamycin (mTOR) inhibitors, and fibroblast growth factor receptor (FGFR) inhibitors (286, 287).

High phosphate intake has been associated with an increased risk of developing several types of solid tumors. In animal models, phosphate ions promote cell cycle progression and lung cancer formation through the AKT (or protein kinase B, PKB) pathway; however, limited information is available for other types of epithelial cancer (288). Overall, the available evidence is scarce and mainly derives from preclinical studies.

### 4.3 Magnesium

Magnesium body concentration is primarily regulated by intestinal absorption and renal reabsorption (the main regulator in the long term). Magnesium is a cofactor in all reactions involving ATP transfer and participates in many vascular and metabolic processes. It is involved in bone mineralization, maintains electrolyte homeostasis inside cells, and has an immunomodulatory function by regulating NF-kB activation and cytokine production (4, 289). Magnesium absorption occurs in the small intestine and is enhanced by vitamin D and carbohydrates. The recommended daily intake for adults is 400–420 mg for males and 310–320 mg for females (4, 290). Foods rich in magnesium include leafy vegetables, nuts, legumes, whole grains, fruits, and fish.

Patients with mild hypomagnesemia (0.7–0.75 mmol/L) may experience fatigue, weakness, tetany, and nystagmus; when magnesium concentration falls below 0.7 mmol/L, tonic–clonic seizures, arrhythmias, and delirium can occur (291). Mild asymptomatic hypomagnesemia can be treated with a diet rich in magnesium. Chronic hypomagnesemia can be addressed with oral supplementation using magnesium gluconate tablets. In cases of severe symptomatic hypomagnesemia, intravenous administration of magnesium sulfate is required (291).

Patients diagnosed with cancer frequently experience hypomagnesemia related to various causes: GI resection, vomiting, diarrhea, steatorrhea, and diabetes mellitus often lead to magnesium deficiency. Moreover, some medications commonly used by oncologists, such as loop diuretics, PPIs, antiacids, and antibiotics (i.e., aminoglycosides), are associated with hypomagnesemia. Cisplatin typically causes mild hypomagnesemia due to renal magnesium wasting, which can occur 3 weeks after the first administration and whose incidence increases with cumulative dose (292–294). Furthermore, cetuximab and panitumumab, two anti-epidermal growth factor receptor (EGFR) monoclonal antibodies frequently used in the treatment of colorectal cancer, directly affect kidney convoluted tubules, leading to renal magnesium wasting (291, 295).

The role of magnesium in tumorigenesis is controversial. It appears to exhibit a dual role, depending on cellular context, concentration, and tumor stage (296, 297). In murine models, magnesium has demonstrated both anti-tumor activity, blocking tumor growth at its primary site, and pro-tumor activity, promoting metastatic colonization. On one hand, adequate magnesium levels support genomic stability, DNA repair, and apoptosis, thereby exerting a protective role against carcinogenesis (298–301). Magnesium also plays a role in modulating cellular immunity and T-cell mediated cytotoxicity, and observational data have indicated an association between its plasma concentration and better outcomes in patients treated with immunotherapy (302, 303). Some evidence links magnesium deficiency to chronic inflammation and increased cancer risk; for example, numerous studies suggest that magnesium has a protective role against breast, colon, and liver cancer (290, 304–306). Magnesium intake has been associated with longer survival in breast cancer patients, particularly in post-menopausal women and those with high calcium/magnesium intake (307). Adequate magnesium intake, in combination with sufficient vitamin D status, was associated with lower mortality in colorectal cancer patients (305, 308). A meta-analysis of prospective studies found an association between dietary magnesium intake and a lower risk of all-cause and cancer mortality, but no associations were found with supplemental intake (309).

On the other hand, magnesium is essential for cell proliferation and may enhance tumor cell growth, especially considering that magnesium transporters like TRPM7 are overexpressed in several solid tumors (310). The TRPM family participates in tumorigenesis by regulating calcium ion balance, cellular oxidative phosphorylation levels, and influencing ROS generation within mitochondria (298, 311). For example, TRPM8 and TRPM2 are significantly overexpressed in prostate cancer, TRPM5 in lung cancer, and TRPM2 and TRPM7 in breast cancer (312). Regarding the SLC family of transport proteins, high SLC41A3 expression has been correlated with poorer prognosis in HCC patients (313). A mutation in the MAGT1 gene, which codes for a magnesium transporter, leads to impaired T-cell activation and an increased risk of hematologic malignancies (314). MAGT1 overexpression is associated with more aggressive colorectal and breast cancer (315, 316).

The overall effect is likely determined by the tumor microenvironment, the expression of magnesium transporters, and the host immune state, although a clear role has yet to be defined (314).

Finally, magnesium supplementation as a preventive measure for patients developing hypomagnesemia during treatment with anti-EGFR antibodies may help mitigate the deficit and prevent associated arrhythmias (317). Additionally, magnesium intake during chemotherapy has been associated with a lower prevalence of chemotherapy-induced peripheral neuropathy (308).

### 4.4 Sodium

Sodium serum concentration primarily reflects water balance and determines extracellular fluid volume and blood pressure. Osmotic pressure can be rapidly regulated by hypothalamic osmoreceptors through increased synthesis of antidiuretic hormone (ADH) and the sensation of thirst. Long-term volume regulation is controlled by arterial blood volume sensors (carotid and aortic baroreceptors, renal afferent arteriole), which regulate the renin–angiotensin–aldosterone system and renal reabsorption. Sodium also plays a role in nerve signal transmission and muscle contraction through Na+/K+ ATPase pumps. It is mostly absorbed in the small intestine and colon. An adequate daily intake of sodium is 1,500 mg (4, 318). Main food sources include processed foods and added salts.

Sodium deficiency is usually detected in older, hospitalized patients. Symptoms of hyponatremia include nausea, vomiting, headache, confusion, lethargy, and coma. To identify the cause of hyponatremia, it is essential to assess the patient's volume status, clinical history, concomitant medications, and serum levels of potassium, glucose, creatinine, blood urea nitrogen, and bicarbonate. Additionally, adrenal insufficiency must be excluded (319). Regarding treatment, it is important to differentiate acute hyponatremia (onset less than 48 h) from chronic hyponatremia (onset >48 h or unknown). Acute hyponatremia must be treated rapidly, especially if symptomatic, with an intravenous bolus of 100–150 mL of hypertonic saline solution, measuring serum sodium between infusions if more than one bolus is required (320). In the case of chronic hyponatremia, treatment is variable: when sodium is 120–129 mEq/L and the patient is mildly symptomatic or asymptomatic, it is sufficient to discontinue any drugs potentially causing hyponatremia, manage the underlying cause of low sodium, and limit oral or intravenous intake of water. If sodium is below 120 mEq/L, a continuous intravenous infusion is required. Once an appropriate serum sodium concentration is reached, subsequent therapy for chronic hyponatremia can include fluid restriction, the use of loop diuretics and oral urea (in patients with SIADH [syndrome of inappropriate antidiuretic hormone secretion)], oral salt tablets, or vasopressin receptor antagonists (319).

Cancer patients frequently experience hyponatremia, which can be cancer-related or induced by anti-tumor therapies. GI losses, the use of diuretics, hemorrhages, and endocrine impairments such as adrenal insufficiency, ACTH (adrenocorticotropic hormone) deficit, or severe hypothyroidism are common causes of low sodium levels. Moreover, SIADH is a typical paraneoplastic syndrome associated with hyponatremia (321).

With regard to its possible carcinogenic role, high intake of salty foods may increase the risk of developing gastric cancer (318). No prospective randomized trials are available on this matter.

At a preclinical level, hyponatremia has been associated with reduced cell adhesion and increased proliferation in cancer cell lines (322). Numerous studies have investigated the role of voltage-gated sodium channels (VGSC), and their expression, which leads to higher intracellular sodium levels, is linked to increased cell motility, proliferation rate, and metastatic potential (323–326). Implicated mechanisms include upregulation of molecular pathways involved in oxidative stress, aberrant calcium signaling, silencing of transcription factors and histone deacetylases, and deregulation of cytoskeleton-associated proteins. In particular, VGSC appear to be overexpressed in the podosomes (called invadopodia), which enable cancer cell invasiveness (323). Several preclinical studies have tested the efficacy of VGSC-inhibiting drugs on cancer cells (327, 328). The prescription of certain VGSC-inhibiting drugs already in clinical use, such as antiepileptic drugs and antiarrhythmic agents, has been associated with reduced cancer incidence and improved survival in retrospective studies (329, 330). Additionally, a randomized trial showed a benefit in disease-free survival in breast cancer patients receiving presurgical peritumoral injection of lidocaine, which is also a VGSC inhibitor (331). Further clinical studies involving VGSC inhibitors are awaited.

### 4.5 Potassium

In potassium homeostasis, intra-/extracellular redistribution is the main rapid form of physiological regulation; for example, a higher intracellular intake can be driven by adrenergic stimulation, insulin, or alkalosis. Long-term regulation occurs through the kidneys, with active secretion in the late distal nephron. Potassium plays a role in maintaining water balance within cells, favors depolarization of nerve tissues and muscle contraction, is involved in ATP production, and maintains acid–base equilibrium (4). It is primarily absorbed in the small intestine, and an adequate daily intake of potassium is 2,300 mg for women and 3,000 mg for men aged 14–18 years, 2,600 mg and 3,400 mg, respectively, after age 19 (332). Foods rich in potassium include fruits, legumes, vegetables, and almonds (4).

Hypokalemia is common in hospitalized patients treated with diuretics and in individuals suffering from inflammatory bowel diseases. Symptoms of hypokalemia include fatigue, muscle weakness, constipation, and impaired cardiac contractility with irregular heart rate. Severe hypokalemia occurs when potassium levels are below 2.5 mEq/L (4, 332). Treatment of mild hypokalemia consists of oral supplementation, with a daily dose of 60–80 mEq of potassium chloride. In cases of chronic refractory potassium deficiency, the addition of a potassium-sparing diuretic can be considered. Symptomatic patients with severe hypokalemia must receive intravenous potassium chloride at a dose of 20 mEq every 2–3 h; alternatively, they can receive oral potassium chloride at a dose of 40 mg 3–4 times a day (333, 334).

Patients with a cancer diagnosis often experience hypokalemia due to various mechanisms. Inadequate potassium intake is typical of patients with anorexia, diarrhea, or bowel malabsorption; some neuroendocrine tumors may produce substances that induce diarrhea (i.e., carcinoid syndrome due to serotonin production). Moreover, other tumors may produce and secrete ectopic ACTH, which causes high blood cortisol levels and consequent urine potassium losses. Concomitant drugs frequently used in oncology patients, such as thiazide diuretics, insulin, granulocyte growth factor, or glucocorticoids, may induce hypokalemia through renal loss or via rapid potassium redistribution inside cells. Furthermore, many oncological drugs are associated with hypokalemia: cisplatin, ifosfamide, anti-EGFR agents, mTOR inhibitors, eribulin, and abiraterone (333).

The role of potassium in carcinogenesis is still not fully understood. However, many potassium channels located in cell membranes contribute to cancer proliferation. For example, in breast cancer, KCNMA1, KCNJ3, KCNN4, and KCNK9 are associated with estrogen receptor expression and lymph node and brain metastases; KCNMA1 is also overexpressed in hormone-sensitive prostate cancer cells. Additionally, KCNQ1 may be expressed by lung tumors and promotes cell proliferation and resistance to hypoxia. KCNH2 plays a role in colorectal cancer, favoring cell proliferation and migration. Furthermore, its overexpression in squamous cell carcinoma of the esophagus, as well as in pancreatic and gastric cancer, has a negative prognostic role (333). Prospective clinical studies are still awaited.

### 4.6 Chloride

Chloride is the main extracellular anion, and its concentration is primarily regulated by kidney excretion. Chloride has several functions in the human body: it maintains pH balance, regulates the quantity of fluids and nutrients inside and outside cells, stimulates gastric parietal cells to release H+ protons, and promotes CO2 transport by red blood cells from tissues to the lungs. Chloride is absorbed in the small intestine, and the recommended daily intake is 2,300 mg for people aged 14–50 years, 2,000 mg for people aged 51–70 years, and 1,800 mg for people aged 71 years or older (4, 335). Cooking salt is the main source.

Hypochloremia is often associated with low serum sodium and/or high serum bicarbonate. Typical symptoms of chloride loss include dehydration, fatigue, shortness of breath, confusion, nausea, and vomiting (336). The treatment of hypochloremia depends on the presence of other electrolyte alterations. If associated with low serum sodium, treatment consists of correcting hyponatremia with intravenous infusion of saline solution. The presence of high serum bicarbonate could indicate chronic respiratory acidosis [typical of chronic obstructive pulmonary disease (COPD)] or metabolic alkalosis (secondary to vomiting, use of thiazide diuretics, or excess mineralocorticoids) (336).

Patients with a cancer diagnosis may experience hypochloremia; in particular, cancer- or CT-related diarrhea and vomiting can cause chloride deficiency (337, 338). Lung cancer patients frequently suffer from COPD, with chronic respiratory acidosis that may lead to hypochloremia (339–341). Moreover, kidney impairment, the use of medications like diuretics and glucocorticoids, or ectopic production of ADH by tumor cells can all be related to hypochloremia (333, 342–344).

As mentioned above regarding sodium, a high intake of salty foods may increase the risk of developing gastric cancer (318); the level of evidence on the oncoprotective/oncogenic role of chloride is limited. Several chloride transporters have been described as overexpressed in solid tumors. TMEM16A is frequently overexpressed in epithelial cancers and contributes to multiple biological functions of cancer cells (345). TMEM206 plays a role in mediating acid-induced cell death as well as cell proliferation and migration, and its regulation is dependent on p53 through a p21-dependent mechanism (346). ANO1 is another calcium-activated chloride channel that plays a central role in the proliferation, invasiveness, and apoptosis of various malignant tumors (347, 348). CLIC1 is an intracellular channel whose overexpression in cancer correlates with increased matrix stiffness (through the Wnt/β-catenin/TCF4 signaling pathway) and altered glycolytic metabolism (Warburg effect), favoring tumor proliferation; its expression level has been retrospectively associated with poorer prognosis in pancreatic and lung cancer (349–352). Dysfunction of the CFTR (cystic fibrosis transmembrane conductance regulator) protein is associated with an increased risk of GI tumors, both in patients with cystic fibrosis and in sporadic GI cancers (353).

### 4.7 Iron

Iron concentration is regulated by the hepcidin/ferroportin axis: liver hepcidin binds to tissue ferroportin, causing its internalization, blocking iron export, and therefore lowering serum concentration. Iron is a fundamental cofactor for a large number of enzymes. It is involved in oxygen delivery (hemoglobin, myoglobin), metabolism of foods and drugs, synthesis of biomolecules (niacin, carnitine, procollagen, nitric oxide, DNA, thyroid hormones), electron transport in ATP production, antioxidant functions, and the destruction of bacteria, viruses, and microbes (4, 354).

Absorption mechanisms differ for heme and non-heme iron. For heme iron (around 25% of the total), hydrolysis from hemoglobin/myoglobin is a crucial step before absorption, involving digestion in the gastric and small intestine by proteases. In the enterocytes, iron is released from the heme porphyrin ring to become available for its functions. The absorption of non-heme iron depends on hydrolysis by gastric and small bowel proteases. The recommended daily intake for adults is 8 mg/day for men and post-menopausal women, and 18 mg/day for premenopausal women (4, 354). Heme iron is found in meat and animal foods, while vegetables are rich in non-heme iron.

Iron deficiency may present with anemia, fatigue, pallor, and increased risk of infections. In most cases, it is detected due to anemia; however, alterations in the anemia profile only appear late during the development of iron deficiency. Additionally, plasma ferritin levels can be influenced by concomitant infections or inflammation, which must be excluded (354). Non-heme iron absorption can be inhibited by several dietary factors as well as intraluminal factors (such as excessive alkalinization of the GI tract, malabsorption syndromes, rapid transit time, and absence of digestive juices). Common causes of excessive alkalinization are pharmacological: H2 receptor blockers (famotidine, cimetidine, nizatidine) and PPIs. Other causes of iron deficiency can include infections, surgical procedures associated with damage/resection of the duodenum and proximal jejunum, and renal diseases. In cancer patients, iron loss could also be due to diarrhea, both as a cancer symptom and as a consequence of anti-neoplastic drugs (4, 354). Iron deficiency can be treated with oral supplements, which provide 30–65 mg to 120 mg of (non-heme) iron according to individual needs. If oral supplementation is contraindicated or insufficient, parental iron administration is available as iron dextran.

Iron overload appears to be associated with an increased risk of cancer, particularly HCC, but also colorectal, breast, and prostate cancers (11). Dysregulation of iron metabolism is quite common in cancer cells. An upregulation of transferrin receptors is frequent, leading to increased iron import; transferrin receptors can also influence the transcription of other genes central to carcinogenesis (i.e., *TP53*) and are being studied for their prognostic role and as potential therapeutic targets (355–357). Hepcidin dysregulation is also common in cancer cells: increased synthesis leads to negative regulation of ferroportin and iron export (358, 359). Iron acts as a tumor growth factor and is involved in cell cycle regulation, DNA metabolism, and cellular energy generation. Moreover, excess iron could promote the formation of reactive oxygen species (ROS), which can lead to oxidative stress and DNA damage, with cytokine-regulated inflammatory pathways and a central contribution from innate immune cells in the tumor microenvironment (360, 361). However, in the complex molecular processes that characterize tumorigenesis, iron also plays a central role in ferroptosis, a form of non-apoptotic regulated cell death characterized by the lethal accumulation of lipid peroxidation products and ROS derived from iron metabolism (362, 363). Among the various mechanisms involved in ferroptosis, ferritinophagy is a selective type of autophagy that specifically targets intracellular ferritin for degradation, facilitating iron recycling in cellular processes (363, 364); its dysregulation may be implicated in the pathophysiology of cancer (365). The mechanisms of ferroptosis are not yet fully understood; in fact, it can also favor cancer development due to its potential negative impact on anticancer immunity (366).

Overall, exhaustive clinical data on the oncogenic effect of iron are still lacking, and further studies are awaited, especially in the clinical setting.

### 4.8 Zinc

Zinc concentration is mainly regulated by fecal excretion, which includes both unabsorbed dietary zinc and pancreatic, biliary, and intestinal secretions (enterohepatic cycling). As an enzymatic element, zinc is involved in most metabolic pathways: protein, lipid, nucleic acid, and carbohydrate metabolism. Zinc is also found in many transcription factors, making it fundamental for regulating gene expression. Moreover, zinc contributes to maintaining and stabilizing cell membranes and microtubules (4, 354). Its absorption occurs in the duodenum and upper jejunum. Zinc chelators, such as prostaglandins, glutathione, pancreatic secretions, tripeptides, amino acids, and organic acids, can promote its absorption; conversely, an alkaline environment, as well as other minerals (calcium and iron), decreases its absorption. The recommended daily intake should be 11 mg for adult men and 8 mg for women (4). It can primarily be found in oysters and shellfish, red meat, dairy products, legumes, and eggs.

Zinc deficiency is characterized by GI symptoms (diarrhea, anorexia), lethargy, depression, alopecia, skin alterations (rash, lesions, dermatitis), hypogeusia, vision problems, and impaired protein synthesis, immune function, and wound healing. Moreover, zinc deficiency promotes impaired glucose tolerance. The most common method to assess deficiency is by measuring serum or plasma zinc concentration after fasting (354).

Several circumstances can favor zinc deficiency, such as alcohol consumption, sickle-cell anemia, and trauma. In cancer patients, intestinal malabsorption (i.e., surgical procedures involving the stomach and duodenum, short bowel syndrome, liver failure), diarrhea and intestinal fistulas, and total parenteral nutrition are all conditions associated with a higher risk of deficiency. Additionally, several drugs can be involved, such as thiazide and loop diuretics (which increase urinary excretion) and antiacid drugs (PPIs, H2 receptor blockers). Zinc deficiency can be treated with oral zinc supplementation; the recommended dose is 10–20 mg/day (4, 354).

The role of zinc in cancer is still controversial. It is a structural component of many transcription factors and DNA repair enzymes, including p53, AP-1, and polymerases. Adequate zinc levels are essential for maintaining DNA integrity and preventing mutations. Zinc deficiency impairs DNA repair and increases oxidative DNA damage, potentially contributing to carcinogenesis. Additionally, zinc acts as a cofactor for superoxide dismutase (Cu/Zn SOD) and regulates the expression of metallothioneins, which scavenge ROS (367). These properties help reduce chronic inflammation, a well-known promoter of tumorigenesis. Moreover, zinc can promote apoptosis in malignant cells by influencing mitochondrial membrane potential and regulating caspase activation. It may also modulate intracellular signaling pathways (i.e., MAPK, NF-κB) to inhibit cell proliferation. Zinc is crucial for the normal function of the innate and adaptive immune systems, including cytotoxic T cells and NK cells, both of which play a role in cancer immunosurveillance.

Despite hundreds of studies in the last few decades, no clear recommendations are available for cancer prevention and treatment (368). Zinc deficiency has been associated with an increased risk for several cancers [such as endometrial (369, 370), prostate (367, 368), pancreatic (368), breast (368), gynecological (371) and lung cancer (372)]. However, other studies have indicated that zinc supplementation could be associated with an increased risk of prostate cancer (373). All the evidence available essentially derives from retrospective data; prospective studies are necessary to confirm the oncoprotective role of zinc intake, as well as to exclude a potential oncogenic role of excessive supplementation, considering the associations reported for prostate cancer.

### 4.9 Copper

Copper homeostasis is maintained by coordinated intestinal absorption, hepatic storage, and biliary excretion. It is a fundamental enzyme cofactor involved in iron oxidation, antioxidant processes, ATP production, norepinephrine synthesis, melanin pigment production, biogenic mono-/diamines oxidation, collagen and elastin cross-linking, blood clotting, immune functions, and activation of selected hormones/peptides. Copper from dietary intake is reduced to its cuprous form (Cu^2+^ -> Cu^1+^) and then absorbed in the small bowel. The recommended daily intake for adults is 900 mg/day (4, 354). Main dietary sources include organ meats, nuts and seeds, legumes, and grain products.

Copper deficiency presents with anemia, leukopenia, muscle weakness, fatigue, altered pigmentation of both skin and hair, impaired immune function, alterations in bone and blood vessel/connective tissue, alterations in cholesterol metabolism, and cognitive deficits. Copper deficiency can be diagnosed with a concentration lower than 70 μg/dL (10 μmol/L) or with a serum ceruloplasmin concentration lower than 20 mg/dL. However, ceruloplasmin is an acute-phase reactant protein and is not specific for copper deficiency (354).

Copper deficiency can result from impaired absorption (due to alkaline pH, use of antiacid drugs, or food complexes such as phytic acid or other minerals), conditions promoting copper loss (e.g., nephrosis), or total parenteral nutrition. Oral copper supplementation is an effective way to treat deficiency: the recommended dose is 1.5–3 mg once or twice a day for 3 months. In cases of zinc-induced copper deficiency or in patients receiving total parenteral nutrition, intravenous supplementation is necessary (4, 354).

Copper-dependent cell growth and proliferation, known as “cuproplasia,” has been recently described in the context of tumorigenesis (374). Cuproplasia could be targeted by pharmacological therapy, either inhibiting or promoting copper signaling. Increased copper levels have been observed in several cancer types, such as breast, endometrial, prostate, GI, lung, and thyroid cancer (374). Conversely, copper can also induce cytotoxicity through ROS accumulation and high mitochondrial-dependent energy metabolism, in a process called “cuproptosis” (374, 375). In this context, an association of decreased copper levels and a higher risk of endometrial (369), gynecological (371), and lung cancer (372) has been reported. All the evidence related to cuproplasia and cuproptosis is derived from retrospective studies, with only the association between reduced copper levels and a higher risk of lung cancer stemming from a meta-analysis of retrospective data. Prospective studies are needed to clarify the possible association of copper intake with the risk of solid tumors.

### 4.10 Selenium

Selenium homeostasis is mainly regulated by renal clearance, with excessive selenium excreted as methylated metabolites. It is an essential element for several proteins and enzymes and is involved in many biological processes: selenoprotein synthesis, antioxidant functions, maintenance of cellular redox state, oxidative damage repair, and thyroid hormone synthesis and metabolism. Moreover, it may play a role in muscle cells, neuronal cells, and the endoplasmic reticulum (4, 354). Selenium absorption occurs in the small intestine, with a recommended daily intake for adults of 55 mg/day (4). It can primarily be found in red meat, fish, eggs, nuts, and dairy products.

Selenium deficiency can present with poor growth, loss of hair and skin pigmentation, muscle weakness and pain, cardiomyopathy, whitened nailbeds, and alterations in the immune system and anti-inflammatory response (4, 354). Selenium status can be assessed by serum or plasma concentration, urinary excretion, or whole blood concentration (4, 354). Oral supplementation with 100–200 mg/day is recommended in cases of deficiency.

Selenium deficiency can be caused by intestinal malabsorption or increased loss through diarrhea, which may be symptoms of cancer or can be associated with anti-cancer treatment (including radiotherapy and gastrointestinal surgery) (376, 377). Moreover, an increased risk of deficiency has been reported in patients undergoing total parenteral nutrition (378–380).

Selenium deficiency has been associated with an increased risk of developing various types of cancer, such as prostate, lung, and colon cancer (381, 382), although data are derived solely from retrospective observations. The mechanism involved is likely related to its antioxidant properties, and high serum concentrations appear to be associated with a lower risk of aggressive disease, though evidence is still limited (373). Considering that selenium is essential for thyroid function, the relationship between its concentration and cancer development remains unclear; a meta-analysis found lower serum levels in thyroid cancer patients compared to healthy controls, supporting a possible significant relationship (383).

A randomized trial on selenium and vitamin E supplementation did not demonstrate a reduction in the risk of prostate cancer (384). Another randomized trial evaluated the efficacy of selenized yeast in reducing the risk of recurrence of non-melanoma skin cancer while also assessing its effect on the risk of other types of cancer; total cancer incidence was lower in patients with lower baseline plasma selenium concentrations (especially in males) (385). A meta-analysis on selenium supplementation did not find a reduction in overall cancer incidence and mortality (386).

A randomized phase III trial evaluated selenium supplementation in selenium-deficient gynecological cancer patients receiving adjuvant radiotherapy, and it proved effective in reducing the incidence of grade 2 diarrhea, particularly in patients with a large planning target volume (387, 388).

### 4.11 Chromium

Chromium concentration is primarily regulated by renal clearance. Chromium plays an important role in enhancing insulin action, though its mechanism remains unclear (4, 354). Chromium absorption occurs in the small intestine. The recommended daily intake for adults varies depending on age and gender: 35 mg for men and 25 mg for women aged 50 years or younger, and 30 mg and 20 mg, respectively for older men and women (4). Main dietary sources include fruits, vegetables, grain products, legumes, and meats.

Chromium deficiency may present as peripheral neuropathy, insulin resistance, and weight loss. No specific tests are currently available to accurately assess chromium status (4). There is no consensus on the dosage for chromium oral or intravenous supplementation, with the latter likely exceeding normal dietary absorption levels.

Chromium deficiency is common in hospitalized patients with malnutrition, especially in cases of total parenteral nutrition without appropriate trace element supplementation. Cancer patients may also be at higher risk of deficiency following gastrointestinal tract surgery (4, 354).

The potential oncogenic risk of chromium is associated with its pentavalent (chromium V) and hexavalent (chromium VI) forms, which are toxic and carcinogenic if inhaled, particularly during industrial exposure (354). The IARC has classified chromium VI as a carcinogen, and a correlation between serum chromium levels and lung cancer has been reported (10). Moreover, several studies have investigated the possible carcinogenic role of chromium in gastric cancer, with at least some signals of increased risk noted among workers with high hexavalent chromium exposure (389). A potentially higher occupational risk has been described for several other solid tumors (390).

### 4.12 Iodine

Iodine concentration is primarily regulated by the thyroid through the synthesis of T4 and T3 hormones, which are stored in follicular colloid (the body's main iodine reserve) and whose release is stimulated by TSH via a negative feedback mechanism. Homeostasis is maintained by the tissue effects of thyroid hormones (with peripheral T4/T3 conversion) and urinary excretion. Iodine is a non-metal, essential mineral commonly found in its ion form, called iodide, which is crucial for synthesizing thyroid hormones. The recommended daily intake of iodine is 150 μg for men and women aged 19 years or older (4). Iodide is rapidly absorbed in the stomach and, to a lesser extent, in the duodenum (4). Major dietary sources of iodine include iodized table salt, fish, sea vegetables, eggs, dairy products, and chicken.

Adults with a daily intake below 10–20 μg can develop hypothyroidism due to inadequate thyroid hormone production. To assess iodine deficiency, urinary mean concentration can be measured (4, 391). The consumption of iodized salt, which contains 10–50 mg of iodine per kg of salt, is recommended in cases of iodine deficiency. Additionally, daily oral supplementation with potassium iodide tablets (doses of 100–300 μg) can be recommended (391, 392).

Cancer patients are generally not at higher risk of iodine deficiency. However, anorexia, GI surgery and malabsorption, reduced intake of iodized supplements, and the area of origin (as soil levels of iodine are very low in mountainous regions) may represent risk factors for hypothyroidism.

An anti-proliferative and pro-apoptotic effect of iodine has been reported in preclinical studies on breast and gastric cancer cells (393), suggesting the importance of supplementation in iodine-poor areas. However, additional studies are needed to better understand the effects of iodine on cancer development.

### 4.13 Manganese

Manganese homeostasis is mainly achieved through hepatobiliary excretion. It acts as an enzyme activator and a constituent of metalloenzymes, being involved in several biological functions: synthesis of urea, bone, cartilage, and connective tissue; metabolism of amino acids and carbohydrates; antioxidant activities; and modulation of second-messenger pathways in tissues (4, 354). Manganese absorption depends on dietary intake and occurs in the small intestine. The recommended daily intake is 2.3 mg for men and 1.8 mg for women (4). It is primarily found in grain products, nuts, legumes, green vegetables, and fruits.

Manganese deficiency can present with skin and hair alterations (such as dermatitis, impaired growth of hair and nails, and color changes in hair and beard), nausea and vomiting, ataxia and loss of equilibrium, altered carbohydrate and lipid metabolism, skeletal changes with defects and poor bone formation, impaired reproductive function, and reduced concentrations of clotting proteins (4, 354). Manganese concentration can be assessed in whole blood and plasma/serum. Manganese deficiency is rare and primarily depends on its elimination from the diet (354). There is no consensus on the dosage for oral supplementation, and potential toxicity from intravenous supplementation has raised growing concerns (394). In particular, patients receiving long-term parenteral nutrition are at risk due to the inclusion of manganese in trace element mixtures, which can induce neurotoxicity (395). Close clinical monitoring is recommended for patients receiving parenteral nutrition for more than 30 days, especially those with underlying liver dysfunction, iron deficiency, or alterations in dopaminergic and catecholaminergic metabolism.

Literature on the role of manganese in cancer is still limited. Some evidence regarding its role in the antitumor immune response has been reported, mainly from animal models; this data may suggest an increased efficacy of anti-PD-1 antibodies in combination with manganese intake, as preliminarily assessed in a phase I study (NCT03991559) (396).

### 4.14 Molybdenum

Molybdenum concentration is primarily regulated by urinary excretion. It is a crucial element for several metalloenzymes involved in redox functions. It is absorbed without digestion in the small intestine, and the recommended daily intake for adults is 45 μg (4, 397). Major food sources include legumes, nuts, organ meats, and dairy products.

Molybdenum deficiency is a rare condition, with symptoms associated with genetic defects in enzymes involved in molybdopterin synthesis. Clinical presentation is characterized by seizures, severe developmental delay, and feeding difficulties. Increased blood concentrations of hypoxanthine, methionine, uric acid, and xanthine can typically be evidenced. No validated test is available to assess molybdenum status, and its concentration can be measured in plasma, red blood cells, and urine. There is no consensus regarding oral or intravenous supplementation (4, 397).

Molybdenum is an essential cofactor for several enzymes, such as xanthine oxidoreductase (XOR), which can play a central role in oncogenesis by either catalyzing the metabolic activation of carcinogenic substances or through its products (i.e., free radicals and ROS) (398). XOR appears to have a dichotomous role in tumor biology: XOR products are associated with both induced mutagenesis and cell proliferation, as well as with cell differentiation and apoptosis (399). Low XOR expression has been linked to worse prognosis in several solid tumors (398, 400). Sulfite oxidase (SUOX) is another enzyme associated with carcinogenesis in preclinical models; its expression decreases with the progression of hepatocarcinogenesis, and it may have a prognostic role in curatively resected HCC patients (401). However, high SUOX expression has also been associated with disease recurrence after surgery in prostate and lung cancer (402, 403).

High molybdenum blood levels have been associated with a lower risk of pancreatic cancer, particularly in ever smokers (404). Interestingly, in preclinical studies, a potential anti-tumor role has been evidenced for tetrathiomolybdate, the molybdenum form used to treat Wilson disease, due to its copper-chelating ability; it inhibits angiogenesis and induces copper deficiency through chelation (397). No data are available in the clinical setting. A phase 1b study is ongoing to evaluate the addition of tetrathiomolybdate to chemotherapy and immunotherapy in the adjuvant setting for high-risk triple-negative breast cancer (NCT06134375).

## 5 Culinary medicine

Culinary medicine is an interdisciplinary field that integrates nutrition, medicine, and culinary arts to promote health and manage disease through food (19). This approach uses evidence-based nutritional guidelines paired with practical cooking skills to empower patients to make informed dietary choices that support their treatment and overall wellbeing. Culinary medicine is especially valuable in managing chronic diseases, including cancer, by addressing nutritional challenges and improving patients' quality of life (405).

As a “proof-of-concept” project to support patients with specific nutritional needs, we have developed a gourmet recipe for managing hypokalemia, crafted by a Michelin-starred chef (Table 5). This recipe not only meets potassium requirements but also provides an enjoyable and refined culinary experience. Additionally, we have created a series of recipes targeting hypocalcemia, offering patients flavorful and nutritionally balanced options to help maintain adequate calcium levels (Supplementary Table S1). We decided to initially focus on hypokalemia and hypocalcemia, which are most common in oncologic patients. These culinary interventions aim to enhance dietary adherence and overall health outcomes by blending the art of cooking with the science of nutrition. Our experience so far is limited, and patient feedback remains anecdotal, as the recipes were offered only to a dozen oncologic patients with specific nutritional needs, independent of their cancer diagnosis. However, all the patients who received instructions for the recipes reported high satisfaction levels in terms of feasibility and clinical utility.

**Table 5:** An example of culinary medicine with a gourmet recipe.

**Tender veal with bread sauce, turnip greens, and pumpkin (ingredients for 4 people)**
Tender veal: 4 veal cheeks (250 g each), 1 carrot, 2 celery stalks, 2 yellow onions, 50 g rosemary, 50 g sage, 500 g red wine, 50 g extra virgin olive oil, salt to taste.
1. Clean the veal cheeks, removing any fat and tendons. Sear the cheeks in a pan until golden brown, then transfer them to a deep baking dish.
2. Dice the celery, carrots, and onions. Brown them in a saucepan. Deglaze with red wine and reduce by half.
3. Add the vegetables, liquid, and herbs to the veal cheeks. Add enough water to cover the cheeks.
4. Cover the dish with aluminum foil and bake at 150 °C (300 °F) for about 2.5 h, until the veal is very tender.
5. Strain the cooking liquid and reduce it in a saucepan until it thickens. Use this to glaze the veal cheeks before serving.
Pumpkin sauce: 1 Mantua pumpkin, 50 g rosemary, 50 g sage, 500 g red wine, 50 g extra virgin olive oil, salt to taste.
1. Cut the pumpkin into four wedges, remove the seeds, and season with salt, oil, and herbs.
2. Wrap the pumpkin wedges in aluminum foil and bake at 220 °C (430 °F) for about 45 min, until tender.
3. Discard the herbs and skin. Puree the pulp in a food processor, emulsifying with oil and water (preferably with some vegetable broth) until smooth and creamy.
Bread sauce: 400 g stale bread, 2 liters vegetable broth, 100 g extra virgin olive oil, salt to taste, squid ink to taste.
1. Cut the bread into cubes and toast in a pan until crispy.
2. Soak the bread cubes with vegetable broth and cook until soft.
3. Blend everything in a food processor until creamy. Add squid ink while blending, just enough to give the sauce a black color.
Sautéed turnip greens: 1 Kg cleaned turnip greens, 50 g extra virgin olive oil, salt to taste, 2 garlic cloves, unpeeled, 1 hot chili pepper.
1. Boil the turnip greens in salted water for 2 min, then cool them in ice water. Drain well and squeeze to remove as much water as possible.
2. In a saucepan, brown the garlic and chili pepper in olive oil. Add the turnip greens and sauté until they absorb the flavors.
3. Shape the turnip greens into four disks by pressing them lightly.
Finishing
1. Place a disk of sautéed turnip greens on each plate.
2. Top with a veal cheek.
3. Surround the veal cheek and turnip greens with two circles of pumpkin sauce and bread sauce.
4. Glaze the veal cheek with the hot reduction sauce before serving.
Nutritional value per serving: Energy 1,62 Kcal, total protein 68 g, total fat 72 g, saturated fatty acids 12 g, monounsaturated fatty acids 48 g, polyunsaturated fatty acids 7 g, total carbohydrates 101 g, simple sugars 30 g, total dietary fiber 6 g, phosphorous 955 g, potassium 3,124 mg.

## 6 Discussion

While the literature is full of evidence regarding the role of micronutrients in cancer prevention and the importance of managing deficiencies in oncologic patients, a major challenge is posed by the quality and heterogeneity of the available data. In fact, the literature is still too limited to draw firm conclusions on many topics, as it is based primarily on observational evidence and only minimally on interventional studies, with a scarcity of randomized controlled trials. Consequently, current clinical guidelines are sometimes heterogeneous or inconsistent due to the limitations of the available scientific evidence.

These considerations are even more central given the data regarding the possible application of vitamins or minerals in a therapeutic setting. With the exception of the firmly established role of ATRA in acute promyelocytic leukemia, no conclusive evidence is available about the potential therapeutic application of addressing micronutrient deficiencies and personalized nutritional interventions.

This represents a limitation for our work and the conclusions that can be drawn from it, particularly for some specific micronutrients for which the oncoprotective or pro-oncogenic role remains unclear. Further interventional trials are definitely needed.

Another limitation is that our review has a narrative nature, which carries an inherent potential for selection bias.

## 7 Conclusions

This comprehensive review underscores the essential role of vitamins and minerals in cancer prevention and management. Micronutrient deficiencies are prevalent among cancer patients due to factors such as the disease itself, treatment side effects, reduced dietary intake, and malabsorption. Addressing these deficiencies through dietary modifications or supplementation has the potential to improve patients' outcomes by strengthening immune function, mitigating treatment-related side effects, and enhancing overall quality of life.

Future research should aim to clarify the specific mechanisms by which these micronutrients impact cancer biology and assess the therapeutic potential of personalized nutritional interventions. Integrating these insights into clinical practice could enable clinicians and dietitians to develop more effective patient care strategies, ultimately optimizing treatment and survivorship outcomes for cancer patients.
