# Supplementary material for: Vitamins and minerals and their role in cancer: a comprehensive review
Source: Front Nutr. 2026 Jan 12;12:1686777. doi: 10.3389/fnut.2025.1686777 (PMC12832475; doi:10.3389/fnut.2025.1686777)
Supplement: Supplementary file 1 [file Table_1.docx]

**Table S1.** Recipes to manage hypocalcemia.

| **BREAD WITH RICOTTA, DRIED FIGS AND PISTACHIO**  **Ingredients for 1 person:** 50 g whole wheat bread (1 medium slice); 50 g cow or goat ricotta; 1 tablespoon (Tbsp) Pistachio grain; 2 dried figs; cinnamon to taste.  **Procedure:**   1. Slightly toast the bread. 2. Spread the ricotta on the bread. 3. Cut dried figs and place them on the ricotta. 4. Finish the plate with pistachio grains and cinnamon.  \| **Ingredients** \| **Kcal** \| **CHO** \| **Sugars** \| **PRO** \| **FAT** \| **Fiber** \| **Calcium** \| \| --- \| --- \| --- \| --- \| --- \| --- \| --- \| --- \| \| Whole wheat bread (50 g) \| 112 \| 24.2 \| 1 \| 3.8 \| 0.7 \| 3.3 \| 12.5 \| \| Cow ricotta (50 g) \| 73 \| 1.8 \| 1.8 \| 4.4 \| 5.5 \| / \| 144 \| \| Pistachio grain (1 Tbsp~ 10 g) \| 60 \| 0.8 \| 0.3 \| 1.8 \| 5.6 \| 1.1 \| 13.1 \| \| Dried fig (2~20 g) \| 51.2 \| 11.6 \| 11.6 \| 0.7 \| 0.5 \| 2.6 \| 37.2 \| \| **TOTAL** \| 296.2 \| 38.4 \| 14.7 \| 10.7 \| 12.3 \| 7 \| 206.8 \|   **OVEN-BAKED GRATIN SARDINES**  **Ingredients for 2 people:** 300 g sardines, filleted and halved; 2 Tbsps almond flour; 1 Tbsp extra virgin olive oil; 1 Tbsp breadcrumbs; fresh or dried sage, finely chopped, to taste.  **Procedure:**   1. Preheat the oven to 180°C (fan-forced). 2. Rinse the filleted sardines and pat them dry with paper towels. 3. In a bowl, mix the almond flour, breadcrumbs, sage, and olive oil. 4. Arrange the sardines on a baking sheet lined with parchment paper. 5. Sprinkle the gratin mixture over the sardines, using your hands to help distribute it evenly. 6. Bake in the oven for about 20 minutes.  \| **Ingredients** \| **Kcal** \| **CHO** \| **Sugars** \| **PRO** \| **FAT** \| **Fiber** \| **Calcium** \| \| --- \| --- \| --- \| --- \| --- \| --- \| --- \| --- \| \| Sardine (150 g) \| 194 \| 2.3 \| / \| 31.2 \| 2.8 \| / \| 50 \| \| Almond flour (1 Tbsp~ 10 g) \| 60 \| 0.5 \| 0.4 \| 2.2 \| 5.5 \| 1.3 \| 24 \| \| Extra olive oil (1/2 Tbsp~5 g) \| 45 \| / \| / \| / \| 5 \| / \| / \| \| Breadcrumbs (1/2 Tbsp ~ 7 g) \| 25 \| 5.5 \| 0.04 \| 0.7 \| 0.15 \| 0.2 \| 8 \| \| **TOTAL** \| 324 \| 8.3 \| 0.44 \| 34.1 \| 13.5 \| 1.5 \| 82 \|   **LEGUME PASTA WITH BROCCOLI RABE**  **Ingredients for 2 people:** 160 g legume pasta (chickpea/lentil/pea); 300 g broccoli rabe; 8-10 olives in oil, drained; 1 clove of garlic or onion; 1 Tbsp extra virgin olive oil; 2 Tbsps breadcrumbs.  **Procedure:**   1. Boil and/or sauté the broccoli rabe in a pan with a clove of garlic, the drained olives, and a tablespoon of extra virgin olive oil. 2. Meanwhile, boil the lightly salted water. 3. Toast the breadcrumbs in a pan with spices of your choice (e.g., chili pepper). 4. Drain the pasta and sauté it in the pan with the broccoli rabe. 5. Serve the pasta topped with the toasted breadcrumbs.  \| **Ingredients** \| **Kcal** \| **CHO** \| **Sugars** \| **PRO** \| **FAT** \| **Fiber** \| **Calcium** \| \| --- \| --- \| --- \| --- \| --- \| --- \| --- \| --- \| \| Chickpea pasta (80 g) \| 269 \| 37.7 \| 2.2 \| 15.8 \| 4.8 \| 10.1 \| 114 \| \| Broccoli rabe (150 g) \| 33 \| 3 \| 3 \| 4.4 \| 0.5 \| 4.4 \| 146 \| \| Olive (5 ~ 20 g) \| 28 \| 0.2 \| 0.2 \| 0.2 \| 3 \| 0.6 \| 13 \| \| Extra olive oil (1/2 Tbsp ~ 5 g) \| 45 \| / \| / \| / \| 5 \| / \| / \| \| Breadcrumbs (1 Tbsp ~ 10 g) \| 35 \| 7.8 \| 0.5 \| 1 \| 0.2 \| 0.3 \| 11 \| \| **TOTAL** \| 410 \| 48.7 \| 5.9 \| 21.4 \| 13.5 \| 15.4 \| 284 \|   **SAVORY MUFFINS**  **Ingredients for 2 people:** 1 egg; 50 g chickpea flour; 75 g whole wheat or gluten-free flour; 2 Tbsps extra virgin olive oil; 100 mL soy/almond/cow's milk; a pinch of salt; 100 g black cabbage or other vegetables of choice; 1 Tsp instant yeast.  **Procedure:**   1. Preheat the oven to 175°C (static). 2. In a bowl, mix the egg with the oil and milk. 3. Add a pinch of salt, sifted flour and yeast. 4. Add chosen vegetables. 5. Mix and transfer the batter to muffin molds. 6. Bake for about 20 minutes.  \| **Ingredients** \| **Kcal** \| **CHO** \| **Sugars** \| **PRO** \| **FAT** \| **Fiber** \| **Calcium** \| \| --- \| --- \| --- \| --- \| --- \| --- \| --- \| --- \| \| Chickpea flour (25 g) \| 79 \| 11.7 \| 0.9 \| 5 \| 1.6 \| 3.4 \| 36 \| \| Whole wheat flour (37.5 g) \| 120 \| 25.4 \| 0.8 \| 4.4 \| 0.4 \| 3 \| 11 \| \| Soy milk (50 mL) \| 16 \| 0.4 \| 0.4 \| 1.5 \| 0.9 \| / \| 7 \| \| Extra olive oil (1/2 Tbsp ~ 5 g) \| 45 \| / \| / \| / \| 5 \| / \| / \| \| Egg (1/2) \| 35 \| / \| / \| 4.3 \| 3.2 \| / \| 18 \| \| **TOTAL** \| 295 \| 12.9 \| 2.1 \| 15.2 \| 11.1 \| 6.4 \| 72 \|   **BREAD BRUSCHETTA WITH TOFU CREAM**  **Ingredients for 2 people:** 150 g whole wheat bread; 200 g plain tofu; 2 sun-dried tomatoes in oil; 2 Tbsp extra virgin olive oil; 1 Tbsp tahina; capers, to taste; basil, to taste.  **Procedure:**   1. Toast the bread slices in a pan. 2. Blend the tofu, tahina, sun-dried tomatoes, olive oil, and capers in a mixer. 3. Spread the tofu cream on the bread, then top with basil leaves.  \| **Ingredients** \| **Kcal** \| **CHO** \| **Sugars** \| **PRO** \| **FAT** \| **Fiber** \| **Calcium** \| \| --- \| --- \| --- \| --- \| --- \| --- \| --- \| --- \| \| Whole wheat bread (75 g) \| 168 \| 33 \| 1.5 \| 6.4 \| 1 \| 5 \| 20 \| \| Tofu (100 g) \| 76 \| 1.9 \| 0.6 \| 8 \| 4.8 \| 0.3 \| 350 \| \| Tahina (10 mL) \| 64 \| 1.4 \| / \| 1.9 \| 6 \| 0.8 \| 11.6 \| \| Extra olive oil (1/2 Tbsp ~ 5 g) \| 45 \| / \| / \| / \| 5 \| / \| / \| \| Sun-dried tomato (1 ~ 5 g) \| 14 \| 2.7 \| 1.8 \| 0.7 \| 0.2 \| 0.6 \| 5 \| \| Basil (10 g) \| 3.9 \| 0.5 \| 0.4 \| 0.3 \| 0.1 \| / \| 25 \| \| **TOTAL** \| 370.9 \| 39.5 \| 4.3 \| 17.3 \| 17 \| 6.7 \| 411.6 \|   **SWEET AND SOUR TEMPEH BITES**  **Ingredients for 2 people:** 200 g tempeh; 1 Tbsp balsamic vinegar; 2 Tbsps water; 1 Tbsp low-sodium soy sauce (optional); 1 Tbsp extra olive oil.  **Procedure:**   1. Cut the tempeh into cubes or strips. 2. In a pan, combine the oil, balsamic vinegar, soy sauce, and water with the tempeh. Cook until the sauce thickens, turning the tempeh halfway through cooking.  \| **Ingredients** \| **Kcal** \| **CHO** \| **Sugars** \| **PRO** \| **FAT** \| **Fiber** \| **Calcium** \| \| --- \| --- \| --- \| --- \| --- \| --- \| --- \| --- \| \| Tempeh (100 g) \| 192 \| 7.6 \| / \| 20.3 \| 10 \| / \| 111 \| \| Balsamic vinegar (1/2 Tbsp ~ 5 g) \| 4.5 \| 0.9 \| 0.8 \| 0.3 \| 0 \| / \| 1.5 \| \| Extra olive oil (1/2 Tbsp ~ 5 g) \| 45 \| / \| / \| / \| 5 \| / \| / \| \| Low-sodium soy sauce (1/2 Tbsp ~ 4.5 g) \| 2.5 \| 0.3 \| 0.02 \| 0.4 \| 0 \| 0.03 \| 1.5 \| \| Sesame seed (5 g) \| 29 \| 1.2 \| / \| 0.9 \| 2.5 \| 0.6 \| 49 \| \| **TOTAL** \| 273 \| 10 \| 0.82 \| 21.9 \| 17.5 \| 0.63 \| 163 \| |
| --- | --- | --- | --- | --- | --- | --- | --- | --- | --- | --- | --- | --- | --- | --- | --- | --- | --- | --- | --- | --- | --- | --- | --- | --- | --- | --- | --- | --- | --- | --- | --- | --- | --- | --- | --- | --- | --- | --- | --- | --- | --- | --- | --- | --- | --- | --- | --- | --- | --- | --- | --- | --- | --- | --- | --- | --- | --- | --- | --- | --- | --- | --- | --- | --- | --- | --- | --- | --- | --- | --- | --- | --- | --- | --- | --- | --- | --- | --- | --- | --- | --- | --- | --- | --- | --- | --- | --- | --- | --- | --- | --- | --- | --- | --- | --- | --- | --- | --- | --- | --- | --- | --- | --- | --- | --- | --- | --- | --- | --- | --- | --- | --- | --- | --- | --- | --- | --- | --- | --- | --- | --- | --- | --- | --- | --- | --- | --- | --- | --- | --- | --- | --- | --- | --- | --- | --- | --- | --- | --- | --- | --- | --- | --- | --- | --- | --- | --- | --- | --- | --- | --- | --- | --- | --- | --- | --- | --- | --- | --- | --- | --- | --- | --- | --- | --- | --- | --- | --- | --- | --- | --- | --- | --- | --- | --- | --- | --- | --- | --- | --- | --- | --- | --- | --- | --- | --- | --- | --- | --- | --- | --- | --- | --- | --- | --- | --- | --- | --- | --- | --- | --- | --- | --- | --- | --- | --- | --- | --- | --- | --- | --- | --- | --- | --- | --- | --- | --- | --- | --- | --- | --- | --- | --- | --- | --- | --- | --- | --- | --- | --- | --- | --- | --- | --- | --- | --- | --- | --- | --- | --- | --- | --- | --- | --- | --- | --- | --- | --- | --- | --- | --- | --- | --- | --- | --- | --- | --- | --- | --- | --- | --- | --- | --- | --- | --- | --- | --- | --- | --- | --- | --- | --- | --- | --- | --- | --- | --- | --- | --- | --- | --- | --- | --- | --- | --- | --- | --- | --- | --- | --- | --- | --- | --- | --- | --- | --- | --- | --- | --- | --- | --- | --- | --- | --- | --- | --- | --- | --- | --- | --- | --- | --- | --- | --- | --- | --- | --- | --- | --- | --- | --- | --- | --- | --- | --- | --- | --- | --- |
